# Advancements in Single‐Atom Catalysts: Synthesis, Characterization, and Applications in Sensing Technologies

**DOI:** 10.1002/smsc.202500449

**Published:** 2025-11-10

**Authors:** Ilakeya Subbiah Arivuthilagam, Raghisa Shahid, Md. Mahbubur Rahman, Jae‐Joon Lee

**Affiliations:** ^1^ Research Center for Photoenergy Harvesting & Conversion Technology (phct) Department of Energy & Materials Engineering Dongguk University Jung‐gu Seoul 04620 Republic of Korea; ^2^ Department of Energy Materials Science and Engineering Konkuk University Chungju 27478 Republic of Korea

**Keywords:** biosensor, environmental monitoring, heterogeneous catalysis, single‐atom catalyst, support engineering

## Abstract

Single‐atom catalysts (SACs) have rapidly progressed from early proof‐of‐concept studies to high‐performance sensing platforms. Their atomically dispersed active sites and tunable coordination environments, offer superior catalytic activity and selectivity compared with conventional nanocatalysts. Recent advances in support engineering, spanning carbon nanomaterials, metal oxides, and metal organic frameworks have enabled precise control over SAC composition, electronic structure, and stability under complex operating conditions. This review summarizes the current state of SAC research from three complementary perspectives. First, it compare top‐down and bottom‐up synthesis strategies, emphasizing scalable approaches that preserve single‐atom dispersion. Second, it outlines the characterization techniques, highlighting how advanced spectroscopy, microscopy, and theoretical calculations are integrated to correlate coordination environments with catalytic performance. Third, it discusses emerging sensing applications including biosensing, environmental monitoring, gas and electrochemiluminescence detection, and photoelectrochemical analysis where SAC‐based materials achieve record‐low detection limits. Despite significant advancements, key challenges remain: (i) preventing atom aggregation under harsh electrochemical conditions, (ii) integrating SACs into miniaturized devices, and (iii) establishing standardized metrics that bridge theoretical predictions and practical performance. This review concludes that addressing these issues will advance SACs toward real‐time sensing, with multi‐atom cooperative sites and AI‐assisted catalyst design as promising strategies to unlock their full potential in next‐generation analytical platforms.

## Graphical abstract

This review highlights the emerging dominance of single‐atom catalysts (SACs) over traditional catalysts, providing an in‐depth discussion of various synthesis strategies and advanced characterization techniques. It examines how support materials influence SAC performance and stability, emphasizes their exceptional sensitivity and selectivity in sensing applications, and outlines key challenges and future directions for scalable synthesis and practical integration.

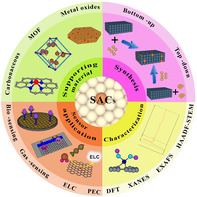

## 1 Introduction

**Figure 1:**
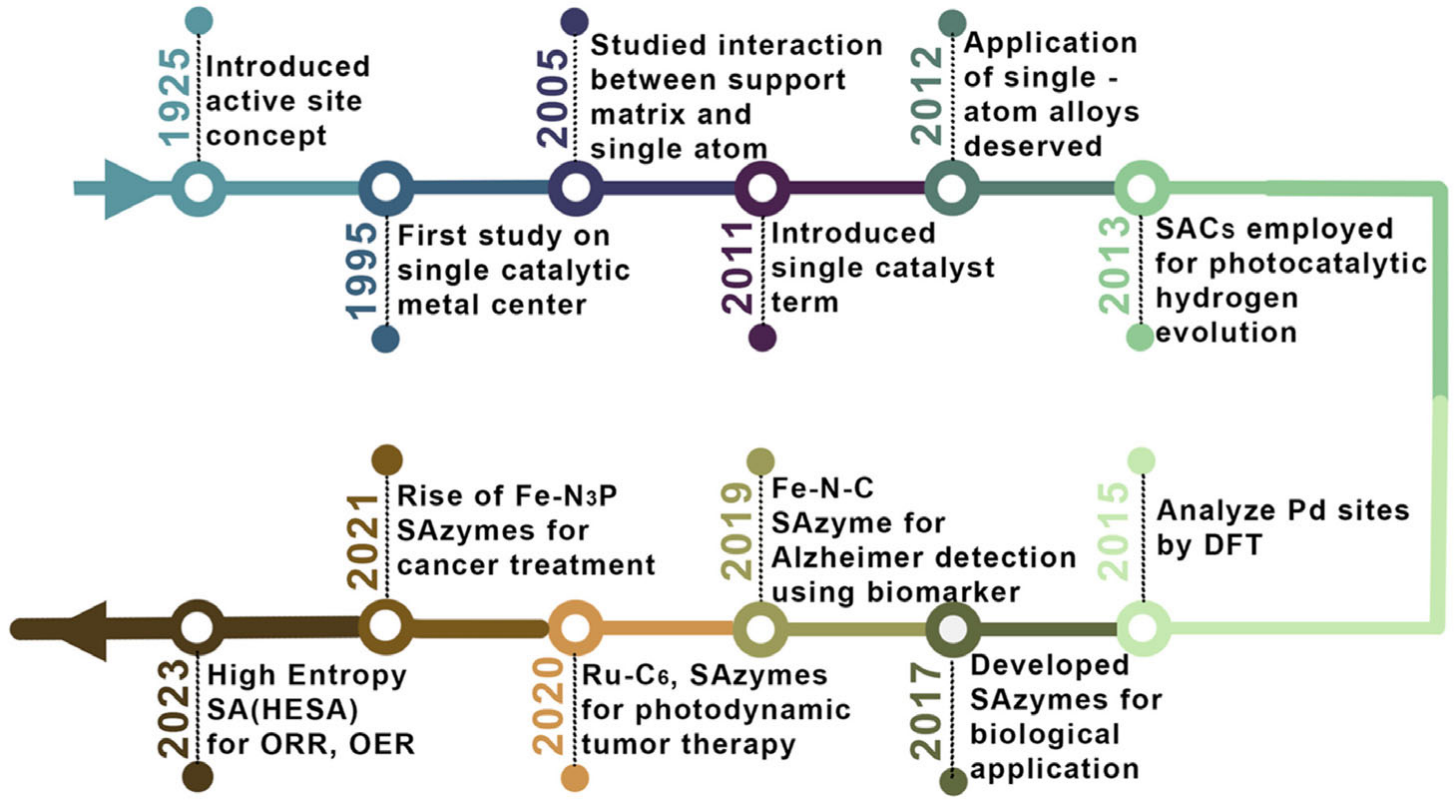
Timeline of the evolution of SACs across diverse applications since 1925.^[^ ^4^, ^18^, ^24^, ^69^, ^152^, ^161^, ^162^, ^163^, ^164^, ^165^, ^166^, ^167^ ^]^

**Figure 2:**
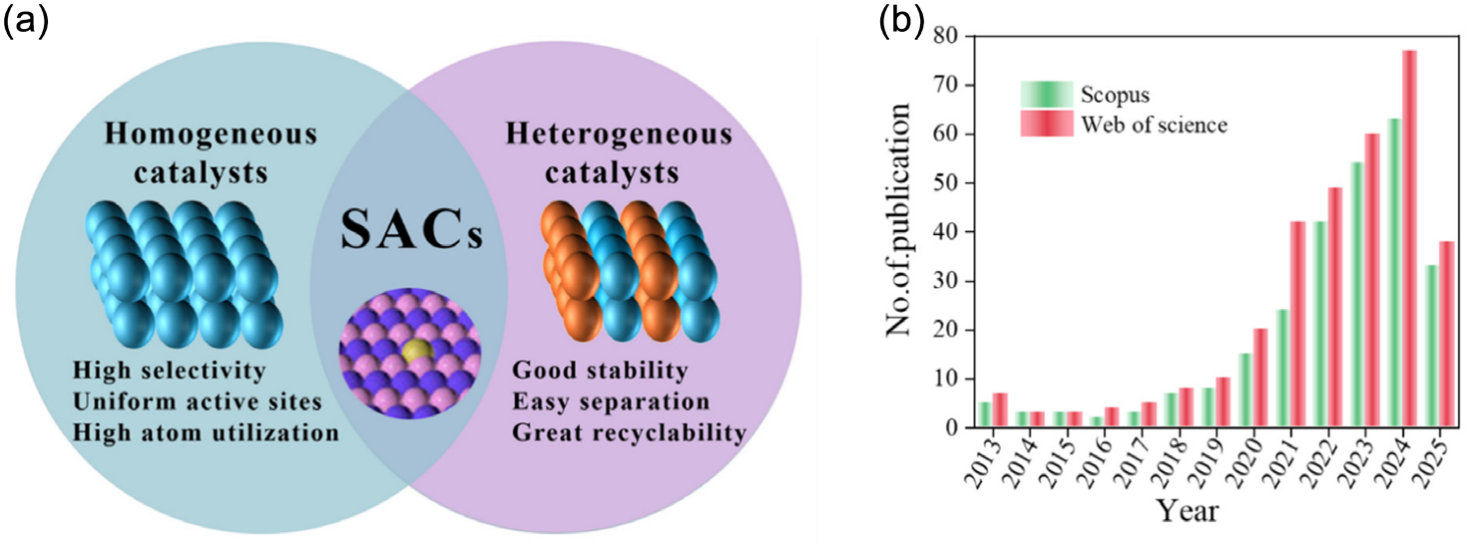
a) Comparison of key characteristics among homogeneous, heterogeneous, and SA catalysts. b) Annual publication trends on “SACs for sensor” from 2013 to 2025. Source: Web of science and Scopus.

Nanocatalysis has advanced rapidly in the past decade as researchers pursue ever‐higher catalytic efficiency, selectivity, and stability.^[^ ^1^, ^2^ ^]^ Nevertheless, conventional nanocatalysts still fall short in applications that demand ultra‐high sensitivity such as environmental, chemical, and biosensing because their structural complexity obscures active‐sites and complicates mechanistic analysis.^[^ ^3^ ^]^ A pivotal breakthrough occurred in 2011 when Zhang et al.^[^ ^4^ ^]^ introduced single‐atom catalysts (SACs): materials in which individual metal atoms are atomically dispersed on solid supports. By maximizing metal utilization and creating uniform active sites, SACs suppress the agglomeration typical of nanoparticle catalysts and often emulate the precision of enzyme centers.^[^ ^5^, ^6^ ^]^ **Figure** **1** charts the accelerating expansion of SACs research across diverse fields. SAC performance is governed by three inter‐connected elements: (i) the supporting matrix, (ii) the metal‐support coordination interface, and (iii) the isolated metal center. Rational design of these factors enables fine control over electronic structure while preserving atomic dispersion. As shown in **Figure** **2**a, the atomic‐scale dispersion of single atoms (SAs) in SACs bridges the gap between homogeneous and heterogeneous catalysis, integrating the benefits of both, namely, the high selectivity and defined active sites of homogeneous systems with the durability and reusability of heterogeneous catalysts. This paradigm shift has driven increased attention toward elucidating the coordination environment of SACs across diverse support materials. For instance, Vile et al.^[^ ^7^ ^]^ demonstrated the effective stabilization of palladium (Pd) SAs on graphitic carbon nitride supports through well‐defined metal–nitrogen (M‐N) coordination motifs, establishing a foundational approach that remains prevalent in SAC design. This study highlighted the critical role of M‐N coordination in simultaneously enhancing catalytic activity and structural stability of isolated metal sites. However, the overall catalytic performance of SACs is strongly influenced by the intrinsic properties of the support, which modulate the stability, electronic structure, and reactivity of the anchored single atoms.^[^ ^8^, ^9^ ^]^

Supporting materials include metal organic frameworks (MOFs) provide well‐defined pores and tunable functional groups that anchor SAs uniformly.^[^ ^10^, ^11^ ^]^ Carbon‐based materials‐graphene, carbon nanotubes, and carbon nitride offer high conductivity and large surface areas, ideal for electrochemical sensors.^[^ ^12^, ^13^, ^14^, ^15^ ^]^ Metal oxides (MO_*x*_) combine thermal robustness with redox activity, furnishing synergistic interactions that enhance catalytic performance.^[^ ^16^ ^]^ Accurately determining oxidation state, coordination geometry, and atom distribution remains challenging. State‐of‐the‐art electron microscopy, X‐ray absorption spectroscopy (XAS), and in situ/operando probes must be integrated with density functional theory (DFT) calculations to build reliable structure‐activity relationships, yet such instrumentation and modeling expertise are not always accessible.^[^ ^17^ ^]^ Computational screening is increasingly indispensable for predicting favorable coordination environments and guiding experiments.

Interest in SAC‐based sensors has grown explosively (Figure 2b). In 2014, SAC‐sensor studies accounted for only ≈1.2% of total SAC publications; by 2024, the share had risen to nearly 28%. This surge reflects SACs ability to endow electrochemical, colorimetric, fluorescence, and photoelectrochemical (PEC) platforms with record‐low detection limits, superior selectivity, and long‐term operational stability.^[^ ^18^, ^19^, ^20^, ^21^, ^22^ ^]^ Representative achievements of iron SACs (Fe‐SACs) include nanomolar detection of dopamine, in‐situ electrochemiluminescent monitoring of heavy‐metal ion of mercury (Hg^2+^) and >90% stability retention in PEC based sensors after 100 h of continuous operation.^[^ ^23^ ^]^

Against this backdrop, the present review critically surveys (i) scalable top‐down and bottom‐up synthesis routes for preserving single‐atom dispersion; (ii) atomic‐level characterization techniques that correlate coordination environment with catalytic behavior; (iii) support‐engineering strategies that stabilize SAs and modulate their electronic properties; and (iv) emerging sensing applications spanning environmental monitoring, biosensing, gas detection, electrochemiluminescence (ECL), PEC, and AI (artificial intelligence) assisted sensor applications. We provide the critical assessment by identifying the key challenges such as stability at high metal loadings, device integration, cost, and mass transport and discussing future directions such as dual‐atom sites, hierarchical porosity, and AI‐based predictive models that could propel SACs toward widespread deployment in next‐generation sensors.

## 2 Synthesis of SACs

Anchoring SACs onto support materials presents significant challenges, particularly in achieving high dispersion without agglomeration. To address these issues, a variety of synthetic strategies have been developed, with the choice of metal precursors playing a critical role in determining the success of these methods. These strategies are generally categorized into top‐down and bottom‐up approaches.^[^ ^24^, ^25^ ^]^ Within these approaches, several methods have been employed to immobilize SAs effectively on various supports.^[^ ^24^, ^26^, ^27^ ^]^ For instance, Bajada et al.^[^ ^28^ ^]^ assessed the sustainability of SACs by synthesizing photocatalysts based on earth‐abundant metals. Their work demonstrated that SACs can be tailored not only for high catalytic performance but also for improved environmental and economic sustainability, marking a step forward in green chemical manufacturing. Extensive research has focused on optimizing these techniques to enhance stability, maximize atomic utilization, and mitigate the inherent difficulties associated with both the synthesis and dispersion of SAs. By refining these strategies, researchers aim to improve the performance of SACs in various catalytic and sensing applications, ensuring high activity and long‐term durability.

### 2.1 Top‐Down Approach

In general, the top‐down approach provides the advantage of preserving the precursor morphology and structural integrity of support materials, allowing for the transformation of bulk materials into atomically dispersed SAs without the need for complex molecular precursors. This approach is particularly well‐suited for large‐scale synthesis due to its ability to maintain the overall structure of the support while effectively dispersing the metal atoms.^[^ ^29^ ^]^ Common techniques used in the top‐down approach include pyrolysis,^[^ ^30^ ^]^ anti‐Ostwald ripening,^[^ ^31^ ^]^ and high‐temperature atom trapping.^[^ ^32^, ^33^ ^]^ These methods have been widely explored to achieve uniform atomic dispersion, while also facilitating the scalability required for industrial applications. Additionally, advanced techniques like mass‐selected soft landing^[^ ^32^ ^]^ have been developed to further enhance the dispersion uniformity of SACs. The versatility of the top‐down approach, demonstrated by its compatibility with a wide range of support materials including carbon‐based substrates, MO_*x*_, and MOFs, significantly broadens the potential for designing diverse catalysts.^[^ ^24^ ^]^ This flexibility makes the top‐down approach a promising pathway for optimizing SACs synthesis across various applications.

#### 2.1.1 Pyrolysis Technique

**Figure 3:**
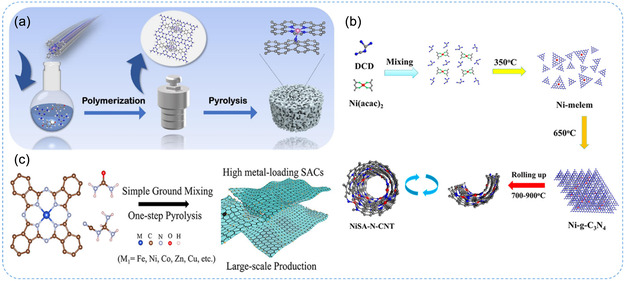
Pyrolysis methods for the synthesis of SACs. a) Schematic illustration of synthesis of Co_1_/NC‐T catalysts. Reproduced with permission from ref. [30] Copyright 2025, Elsevier. b) Scheme for the formation of tubular structured Ni SACs, NiSA‐N‐CNT. Reproduced with permission from ref. [36] Copyright 2018, American Chemical Society. c) Schematic illustration of the synthesis of M_1_‐2D‐NOCs. Reproduced with permission from ref. [37] Copyright 2024, American Chemical Society.

To achieve high loading of SACs, metal atoms can be trapped in situ on nanoporous support materials, effectively preventing their aggregation. In this process, metal precursors are anchored and stabilized on the surface of porous materials through coordination with modified groups on the support.^[^ ^34^ ^]^ These metal precursors are then converted into isolated metal atoms upon pyrolysis. A particularly effective strategy involves synthesizing SACs supported by porous carbon materials, with a focus on nitrogen‐coordinated carbon matrices. In these systems, nitrogen atoms with lone electron pairs act as coordination sites, playing a crucial role in preventing the migration and aggregation of metal atoms. This method helps maintain the atomic dispersion of metal centers, thereby enhancing the stability and catalytic performance of the SACs. Accordingly, Zhang et al.^[^ ^30^ ^]^ reported a highly efficient Co SACs on N‐doped carbon (Co/NC‐850) (**Figure** **3**a). The catalyst was synthesized by in‐situ doping cobalt phthalocyanine into a Schiff base polymer, followed by pyrolysis at high temperatures. The strong confinement of Co‐N motifs within the phthalocyanine rings, coupled with their uniform dispersion throughout the polymer network, significantly enhanced the catalyst's performance. In another report, Wei et al.^[^ ^35^ ^]^ synthesized Fe SACs by pyrolyzing Fe‐doped zeolitic imidazolate frameworks (ZIF‐8). Following this, Pd^2+^ ions were introduced into the Fe SACs matrix via a micropore adsorption technique and subsequently reduced in situ to form Pd nanoclusters (Pd_NC_). The results confirmed that Fe atoms were atomically dispersed within the catalyst. To enhance the peroxidase‐like activity of the Fe SACs, spin state engineering was employed. The incorporation of Pd_NC_, possessing strong electron‐withdrawing characteristics, altered the spin state of Fe (II) in Fe SACs–Pd_NC_ from a low‐spin to a medium‐spin configuration, thereby promoting the heterolytic cleavage of H_2_O_2_. Zhao et al.^[^ ^36^ ^]^ developed a one‐pot pyrolysis method to synthesize nickel single‐atom catalysts (Ni SACs) with an ultrahigh Ni loading (20.3 wt%) on a N‐doped carbon nanotube structure (N‐CNT) (Figure 3b). Extended X‐ray absorption fine structure (EXAFS) analysis confirmed the formation of bamboo‐shaped N‐CNTs that stabilized the Ni atoms through Ni–N_4_ coordination. The resulting NiSA‐N‐CNT catalyst demonstrated significantly enhanced electrochemical performance for the CO_2_ reduction reaction. Butburee et al.^[^ ^37^ ^]^ presented a solvent‐free, one‐step pyrolysis method for synthesizing a range of SACs incorporating various metals (M = Fe, Co, Zn, Cu, Ni, etc.) with high metal loadings (5.2–15.9 wt%) on 2D nitro‐oxygenated carbon (M‐2D‐NOCs) (Figure 3c). The catalysts exhibited a high density of active sites featuring N/O coordination environments, which imparted excellent catalytic activity and stability, as demonstrated in the oxygen reduction reaction (ORR).

Despite its wide applicability and success in achieving high‐loading SACs, pyrolysis‐based synthesis presents inherent challenges that limit its scalability and reproducibility. At elevated temperatures, inconsistent atom dispersion, nanoparticle formation, and metal loss can occur, especially when metal loadings exceed ≈5 wt% without sufficient spatial confinement. Additionally, the method requires precise thermal control and specialized equipment, which can hinder large‐scale production. Batch‐to‐batch variability and the potential for precursor contamination further complicate the process. While advances in precursor design and coordination confinement have partially addressed these limitations, high‐temperature treatments still pose a risk of atom migration and structural degradation. Therefore, ongoing research aims to optimize pyrolysis conditions and develop complementary stabilization strategies to enable scalable, high‐performance SACs with controlled architectures.

#### 2.1.2 Anti‐Ostwald Ripening Method

**Figure 4:**
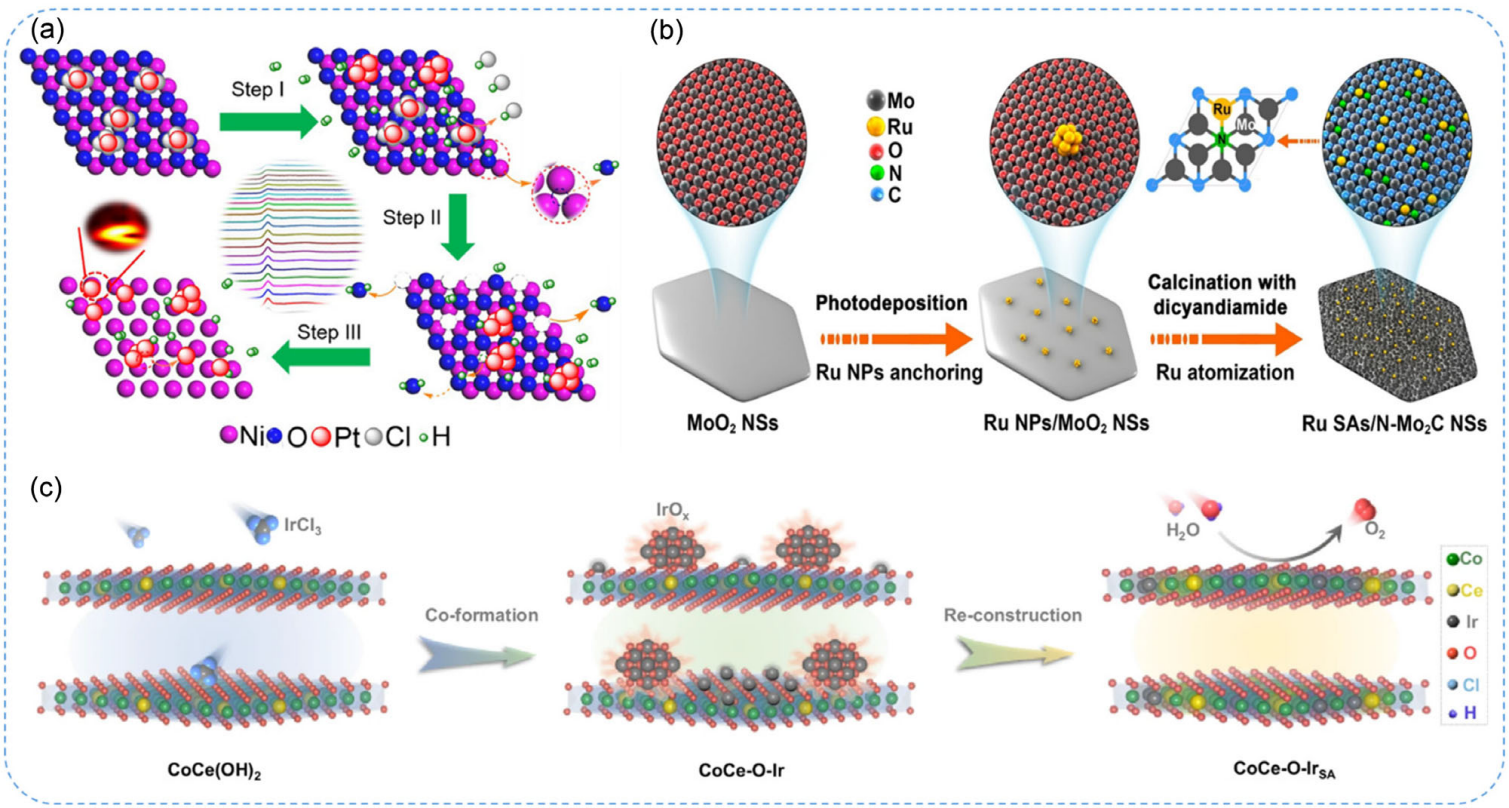
Anti‐Ostwald ripening method. a) Schematic illustration of the formation of Pt SA‐Ni_*x*_. Reproduced with permission from ref. [39] Copyright 2023, Wiley. b) Schematic representation of the synthesis route for Ru SAs/N‐Mo_2_C NSs electrocatalyst. Reproduced with permission from ref. [31] Copyright 2025, Elsevier. c) Synthesis scheme of CoCe–O–IrSA. Reproduced with permission from ref. [40] Copyright 2025, Springer Nature.

Supported SACs often suffer from thermal instability, as high‐temperature treatments during synthesis can cause metal atoms to migrate and aggregate into clusters through Ostwald ripening. In contrast, anti‐Ostwald ripening, a thermodynamically less favorable but achievable strategy, can redistribute metal nanoparticles into atomically dispersed SAs.^[^ ^38^ ^]^ This approach facilitates the formation of stable SACs by pyrolyzing preformed metal nanoparticles on appropriate supports that can trap SAs and suppress their mobility. For instance, Xiao et al.^[^ ^39^ ^]^ developed a stable platinum (Pt) SAC, where Pt atoms were stabilized within highly confined Ni species (Pt‐Ni_*x*_) on a nonreducible Al_2_O_3_ matrix. The catalyst was synthesized via a reduction‐induced anti‐Ostwald ripening strategy, which effectively dispersed Pt SAs (**Figure** **4**a). The formation of isolated Pt atoms was driven by Pt‐Ni_*x*_ metallic bond interactions at high reduction temperatures, as confirmed by in‐situ XAS. In another report, Yu et al.^[^ ^31^ ^]^ tackled the issue of ruthenium (Ru) catalyst sintering by anchoring Ru SAs onto ultrathin N‐doped molybdenum carbide nanosheets (N‐Mo_2_C NSs) using an anti‐Ostwald ripening strategy (Figure 4b). Strong interactions between Ru atoms and the N‐Mo_2_C NSs enable uniform atomic dispersion, confirmed by aberration‐corrected high‐angle annular dark field scanning transmission electron microscopy (AC HAADF‐STEM) and XAFS analyses. Moreover, DFT calculations attribute enhanced activity to synergistic water dissociation and optimized hydrogen adsorption at the Mo‐Ru interface, offering valuable insights for designing efficient SA electrocatalysts. Quan et al.^[^ ^40^ ^]^ introduced an “atomic‐scale self‐rearrangement” strategy using an anti‐Ostwald ripening process under ambient conditions to synthesize high‐density iridium (Ir) SAs embedded in ultrathin CoCeOOH nanosheets (CoCe‐O‐Ir SA) (Figure 4c). Through theoretical calculations, they demonstrated that the Ir SAs enhance electron transfer via strong p‐d‐f orbital coupling, optimize the adsorption of reaction intermediates, and activate the adjacent Co and Ce sites, collectively boosting catalytic performance and thermal stability of SACs. Altogether, compared to conventional SAC synthesis methods, anti‐Ostwald ripening is particularly advantageous for applications requiring high thermal stability. However, its broader adoption for large‐scale production remains constrained by factors such as high material costs, complex synthesis protocols, and limited scalability.

#### 2.1.3 Mass‐Selected Soft‐Landing Technique

The mass‐selected soft‐landing technique is a powerful method primarily employed in academic research for synthesizing supported metal clusters and SACs. Unlike scalable methods such as pyrolysis, it is not suitable for practical applications due to its low throughput. However, it offers exceptional control over the size, composition, and charge state of deposited metal species by using mass‐selected atomic or molecular ion beams.^[^ ^32^ ^]^ When combined with ultrahigh vacuum (UHV) conditions, it allows for fine‐tuning of the support material's structure, enabling the formation of highly uniform and well‐defined catalytic systems. This makes the technique ideal for fundamental studies of metal‐support interactions and cluster size effects at the atomic scale. For instance, Weber et al.^[^ ^41^ ^]^ demonstrated the use of mass‐selected Pt_*n*_^+^ ion deposition under UHV conditions to fabricate size‐selected Pt cluster electrodes (Pt_*n*_, where *n* ≤ 14) on glassy carbon and indium tin oxide supports. These model systems enabled detailed in situ investigations and notably, catalytic activity varied non‐monotonically with cluster size and showed strong correlations or anticorrelations with the Pt 4d core‐level binding energies, underscoring the critical role of electronic structure over geometric factors in determining catalytic performance. Overall, this work highlights the unique capabilities of mass‐selected cluster deposition for probing size‐ and structure‐dependent catalytic phenomena with atomic precision. However, the inherently high cost, complex instrumentation, and low material yield significantly limit its scalability, making it unsuitable for practical industrial applications in heterogeneous catalysis.

#### 2.1.4 High‐Temperature Atom Trapping Method

**Figure 5:**
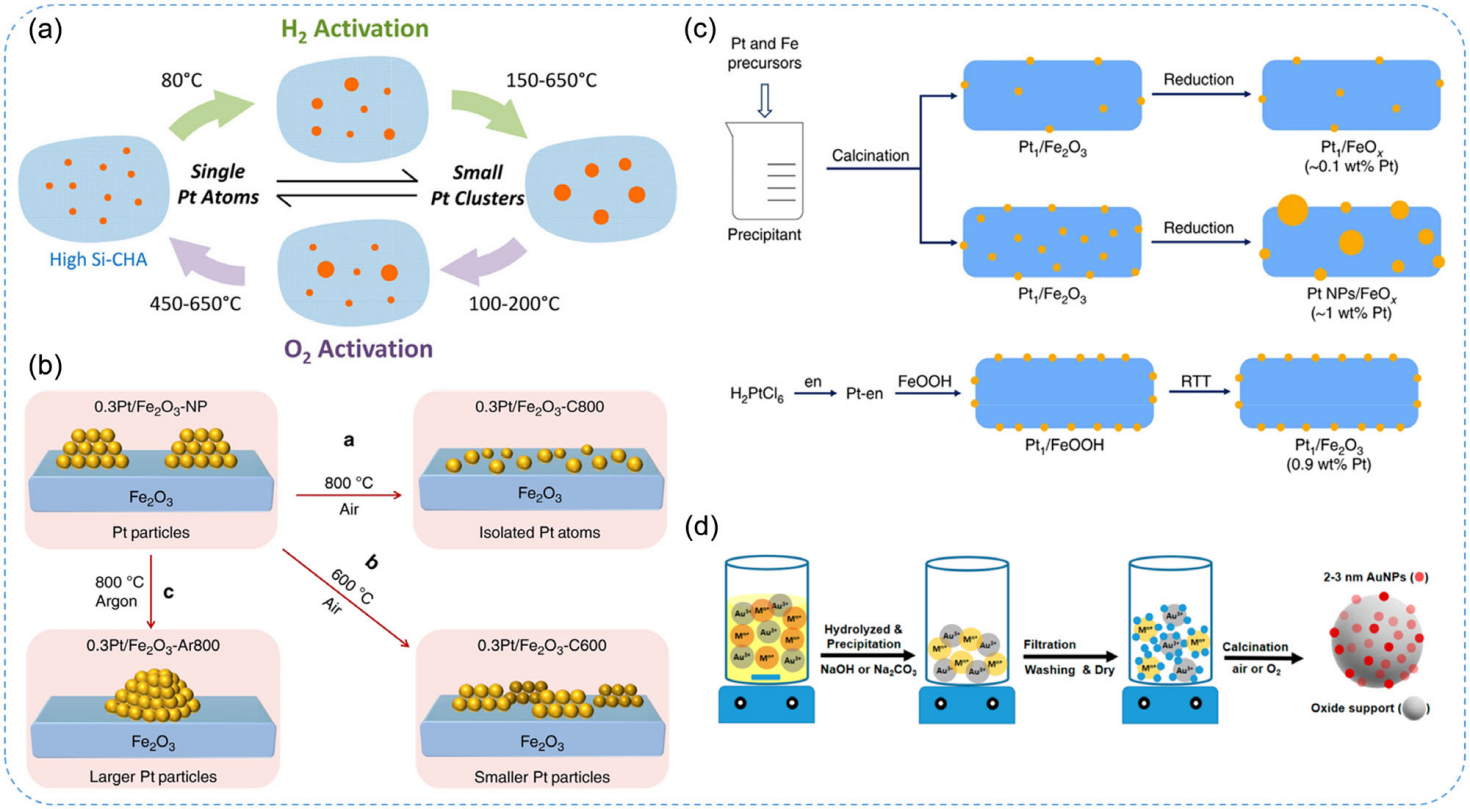
Schematic representation of SACs synthesis using the a,b) high‐temperature atom trapping and c,d co‐precipitation method. a) Illustration of thermally induced restructuring of Pt nanoparticles during the synthesis of Pt‐containing high‐silica CHA materials. Reproduced with permission from ref. [33] Copyright 2016, American Chemical Society. b) Calcination of PtO_2_ under oxygen or inert atmosphere at various high temperatures leads to the formation of Pt SAs under specific conditions. Reproduced with permission from ref. [43] Copyright 2019, Springer Nature. c) Schematic illustration of the synthesis of Pt_1_SA/Fe_2_O_3_ catalysts at RTT conditions. Reproduced with permission from ref. [55] Copyright 2019, Springer Nature. d) General scheme of the co‐precipitation method for SAC synthesis. Reproduced with permission from ref. [57] Copyright 2019, MDPI.

Atom trapping is an emerging top‐down secondary strategy for preparing thermally stable SACs. A key method under this approach is the high‐temperature atom trapping technique, in which metal precursors are oxidized into MO_*x*_ under an oxidative atmosphere at elevated temperatures.^[^ ^42^ ^]^ These MO_*x*_ can then migrate and become anchored onto suitable supports through strong interactions between the metal atoms and specific sites on supports such as oxygen vacancies, terminal hydroxyl groups, or other surface defects which serve as high affinity binding sites, resulting in atomically dispersed metal species. While high temperatures typically promote metal atom aggregation into larger particles, thereby reducing active surface area and catalytic efficiency, this method turns that challenge into an advantage. Moliner et al.^[^ ^33^ ^]^ reported the successful encapsulation of Pt species within highly siliceous chabazite (CHA) zeolites synthesized using N,N,N‐trimethyl‐1‐adamantammonium and a thiol‐stabilized Pt complex, as shown in **Figure** **5**a. Compared with conventional Pt/SiO_2_ or Pt‐loaded Al‐rich zeolites, the Pt/CHA materials exhibit significantly enhanced thermal stability against sintering under industrially relevant environments, including hydrogen, oxygen, and steam. Notably, they maintained structural integrity even at high temperatures (650–750 °C). Advanced characterization using XAS and AC HAADF‐STEM revealed the strong chemical bonding of Pt‐O coordination motifs, between single Pt atoms and small nanoparticles depending on redox conditions, highlighting the dynamic nature of the metal species. These findings underscore the potential of zeolite encapsulation strategies in stabilizing SA and nanoparticle catalysts under harsh conditions. Lang et al.^[^ ^43^ ^]^ demonstrated that isolated Pt atoms can be effectively anchored on supports through covalent metal‐support interactions, either by direct trapping of pre‐deposited atoms or by capturing PtO_2_ species volatilized from nanoparticles during high‐temperature calcination. Both experimental and theoretical analyses revealed that Pt‐O and Pt‐O‐Fe coordination motifs in iron oxide (Fe_2_O_3_) play a key role in anchoring Pt atoms, leading to significantly enhanced catalytic activity compared to conventional Pt nanoparticles. Furthermore, this defect‐free stabilization method is shown to be applicable to nonreducible support through (Fe_2_O_3_) doping, as presented in Figure 5b, offering a versatile and scalable route for developing high‐loading SACs for various industrial catalytic applications. This method represents an effective top‐down strategy for synthesizing thermally stable SACs by exploiting defect‐rich supports to immobilize volatile or mobile metal species. These defects facilitate the formation of stable M‐O and M‐O‐M coordination motifs, which not only anchor isolated metal atoms but also enhance catalytic activity by modulating the local electronic environment.

#### 2.1.5 Sacrificial Template‐Assisted Strategy

Another emerging synthesis strategy employs a temporary sacrificial scaffold (template), such as carbon derivatives, MOF precursor, and layered hydroxide to stabilize and precisely deposited SAs during synthesis.^[^ ^44^, ^45^ ^]^ Following the template removal, atomically dispersed metal sites are anchored onto a permanent support material.^[^ ^44^, ^46^ ^]^ This approach ensures uniform dispersion of SAs, prevents their aggregation, and enhances catalytic activity, while also allows for the creation of complex nanostructures with high surface area and tailored well‐defined porosity.^[^ ^8^, ^47^ ^]^ For instance, Yoon et al.^[^ ^48^ ^]^ developed Co SACs embedded on vanadium oxides nanobelts (V_2_O_5_ NBs) via template assisted hydrothermal synthesis. In this approach, TEMPO‐oxidized cellulose nanocrystal (TCNCs) was mixed with Co precursor, transferred the solution into autoclave and heated at 220 °C for 8 h. During the reaction, TCNCs template was thermally decomposed and promotes the high crystallinity of Co‐V_2_O_5_ NBs. Kim et al.^[^ ^49^ ^]^ reported several strategies of high SAs loading on various support, including metal, MO_*x*_, and perovskites via solution‐mediated process. During the process, Pt SAs on nitrogen doped graphene were destabilized and removed upon exposure to high annealing temperature, whereas Pt SCs on SnO_2_ formed covalent metal‐support interaction and exhibits thermal stability.

Ultimately, this strategy provides significant advantages over other top‐down synthesis routes for SACs. Unlike pyrolysis and high‐temperature atom trapping, it enables precise spatial confinement of metal species, effectively preventing aggregation even at high metal loadings. Compared with the anti‐Ostwald ripening approach, it enables higher metal loadings with better‐controlled active‐site distribution. Moreover, unlike mass‐selected soft landing, which offers atomic precision but suffers from low throughput and poor scalability, the sacrificial template strategy is more cost‐effective and adaptable for large‐scale production. Together, these merits establish it as one of the most practical and versatile methods for synthesizing high‐performance SACs.

### 2.2 Bottom‐Up Approach

A bottom‐up approach refers to constructing structures from the smallest building blocks such as atoms, molecules, or nanoparticles and assembling them into larger, more complex systems. This strategy enables precise control over composition and morphology, and is commonly implemented using techniques such as co‐precipitation,^[^ ^50^ ^]^ impregnation,^[^ ^51^ ^]^ deposition‐precipitation,^[^ ^52^ ^]^ and atomic layer deposition (ALD).^[^ ^53^ ^]^ This process typically involves the adsorption, reduction, and fixation of metal precursors at defect sites on the support material. While these methods are easy to implement, however, they can suffer from the drawback that excessive metal precursors may lead to the aggregation of SAs into clusters, thus compromising atomic dispersion and catalytic performance.

#### 2.2.1 Co‐precipitation Method

Co‐precipitation is a widely employed bottom‐up strategy for synthesizing SACs, wherein metal precursors and support precursors are co‐precipitated from a solution by modulating the pH through the addition of a precipitating agent. This method typically involves incorporating metal precursors such as Ru, Pt, Pd, Ni, Ir, or Cu with support materials^[^ ^50^ ^]^ by introducing precipitating agents like urea or NaOH or by tuning environmental conditions to promote uniform precipitation and atomic‐level mixing. Sun et al.^[^ ^54^ ^]^ reported the synthesis of Ru SACs supported on FeO_*x*_ via a co‐precipitation method, which exhibited outstanding performance in the water–gas shift reaction. Even at an ultra‐low Ru loading of 0.18 wt%, the catalyst achieved specific reaction rates nearly three times higher than those of conventional Ru nanoparticles with a higher loading of 2.0 wt% at 300 °C. Structural analyses revealed that the isolated Ru atoms exhibited strong interactions with the FeO_*x*_ support, forming coordination motifs of Ru–O and Ru–O–Fe, which serve as active sites and significantly enhance catalytic activity. Ren et al.^[^ ^55^ ^]^ studied an effective strategy in which Pt cations are first chelated with ethylenediamine, followed by a rapid thermal treatment (RTT) in an inert atmosphere to remove the ligand. This method enables fine control over the coordination chemistry of Pt SAs supported on Fe_2_O_3_ by simply adjusting the RTT temperature (Figure 5c). In another report, Lin et al.^[^ ^56^ ^]^ synthesized Ir SAs supported on FeO_*x*_ and demonstrated that ≈70% of the total catalytic activity originates from the Ir SAs, identifying them as the dominant active sites. Structural and spectroscopic analyses revealed that the isolated Ir atoms are stabilized through Ir–O and Ir–O–Fe coordination environments, which not only anchor the single atoms but also modulate the electronic structure of the support. These coordination motifs enhance the reducibility of the FeO_*x*_ lattice and promote the formation of oxygen vacancies, which are key to superior catalytic performance. However, stabilizing SAs on the support during the co‐precipitation process requires specific conditions, including elevated temperatures, high reagent concentrations, and precise control over pH and reaction time, as illustrated in the generalized scheme of co‐precipitation in Figure 5d.^[^ ^57^ ^]^ Overall, co‐precipitation offers a straightforward and scalable route to SACs with atomically dispersed metals stabilized by strong coordination motifs such as M‐O and M‐O‐Fe. This strategy improves catalytic activity by enhancing metal dispersion and tuning the support's electronic properties. Nonetheless, challenges remain in precisely controlling atomic loading, preventing agglomeration at higher metal concentrations, and tailoring support chemistry for diverse catalytic applications.

#### 2.2.2 Impregnation Method

**Figure 6:**
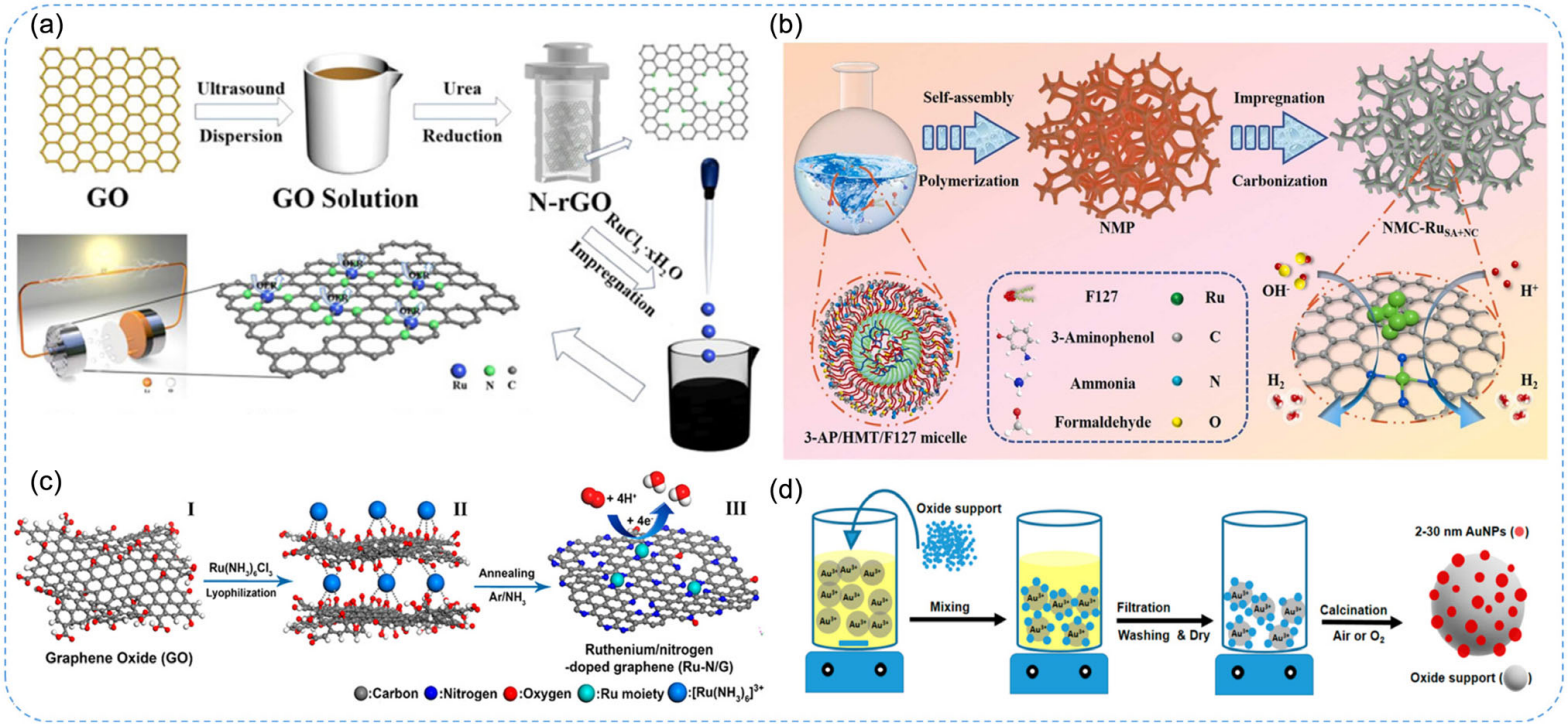
Impregnation method for the synthesis of SACs. a) Schematic illustration of the fabrication of Ru SA on N‐rGO. Reproduced with permission from ref. [58] Copyright 2021, Elsevier. b) Schematic representation of the synthetic process of Ru SA and ultrafine Ru nanoclusters on porous N‐doped carbon (NMC‐Ru_SA+NC_). Reproduced with permission from ref. [59] Copyright 2022, Elsevier. c) Illustration of Ru SAC synthesis using nitrogen doped graphene (NG) as a supporting material and annealed at 750 °C with NH_3_ to synthesize Ru‐N/G‐750. Reproduced with permission from ref. [60] Copyright 2017, American Chemical Society. d) Generalized schematic of the impregnation method. Reproduced with permission from ref. [57] Copyright 2019, MDPI.

In this process, catalytic components are first adsorbed onto the surface of supports in a solution system, followed by drying, calcination, or activation to enhance metal‐support interactions, thereby anchoring SAs onto the support. For instance, Liu et al.^[^ ^58^ ^]^ reported the synthesis of a Ru SACs anchored on N‐doped reduced graphene oxide (N/rGO), where the isolated Ru atoms were stabilized through Ru‐N coordination motifs. Similarly, Yao et al.^[^ ^59^ ^]^ developed a catalyst comprising Ru SAs coupled with porous N‐doped carbon (NMC‐Ru_SA+NC_), synthesized via a simple impregnation method as illustrated in **Figure** **6**a,b. In both cases, the incorporation of N heteroatoms and the presence of structural defects in the support alters the local coordination environment, forming unsaturated Ru‐N_*x*_ sites that acted as “traps” for Ru^3+^ ions, thereby enabling atomic dispersion. Zhang et al.^[^ ^60^ ^]^ reported the synthesis of atomically dispersed Ru on N‐doped graphene (Ru‐NG) via NH_3_ annealing of a Ru‐containing graphene oxide precursor, as demonstrated in Figure 6c. Structural analyses confirmed isolated Ru atoms coordinated with Ru‐N_4_ and Ru‐O‐N motifs, while DFT calculations suggested a Ru‐oxo‐N_4_ configuration as the active site, highlighting the importance of coordination motifs in both stability and activity. Yu et al.^[^ ^61^ ^]^ synthesized an amorphous Al_2_O_3_ support using a hydrothermal method, and subsequently stabilized Fe SAs via a simple wet impregnation process. XAS and HRTEM confirmed that Fe atoms were atomically dispersed and predominantly stabilized through Fe‐O coordination motifs with surface oxygen atoms of Al_2_O_3_. However, this method requires high‐quality carriers with large surface areas, and precise control over factors such as pH, as demonstrated in the conceptual scheme of impregnation method in Figure 6d.^[^ ^57^ ^]^ Overall, the impregnation approach stabilizes isolated atoms through strong coordination motifs like M‐N_*x*_ and M‐O. By leveraging heteroatom doping, structural defects, and high‐surface‐area supports, it enables stable atomic dispersion and tunable catalytic activity. Nonetheless, its dependence on the nature of supports and stringent synthesis control underscores the need for further optimization to achieve reproducible, scalable, and high‐performance SACs.

#### 2.2.3 Atomic Layer Deposition (ALD) Technique

**Figure 7:**
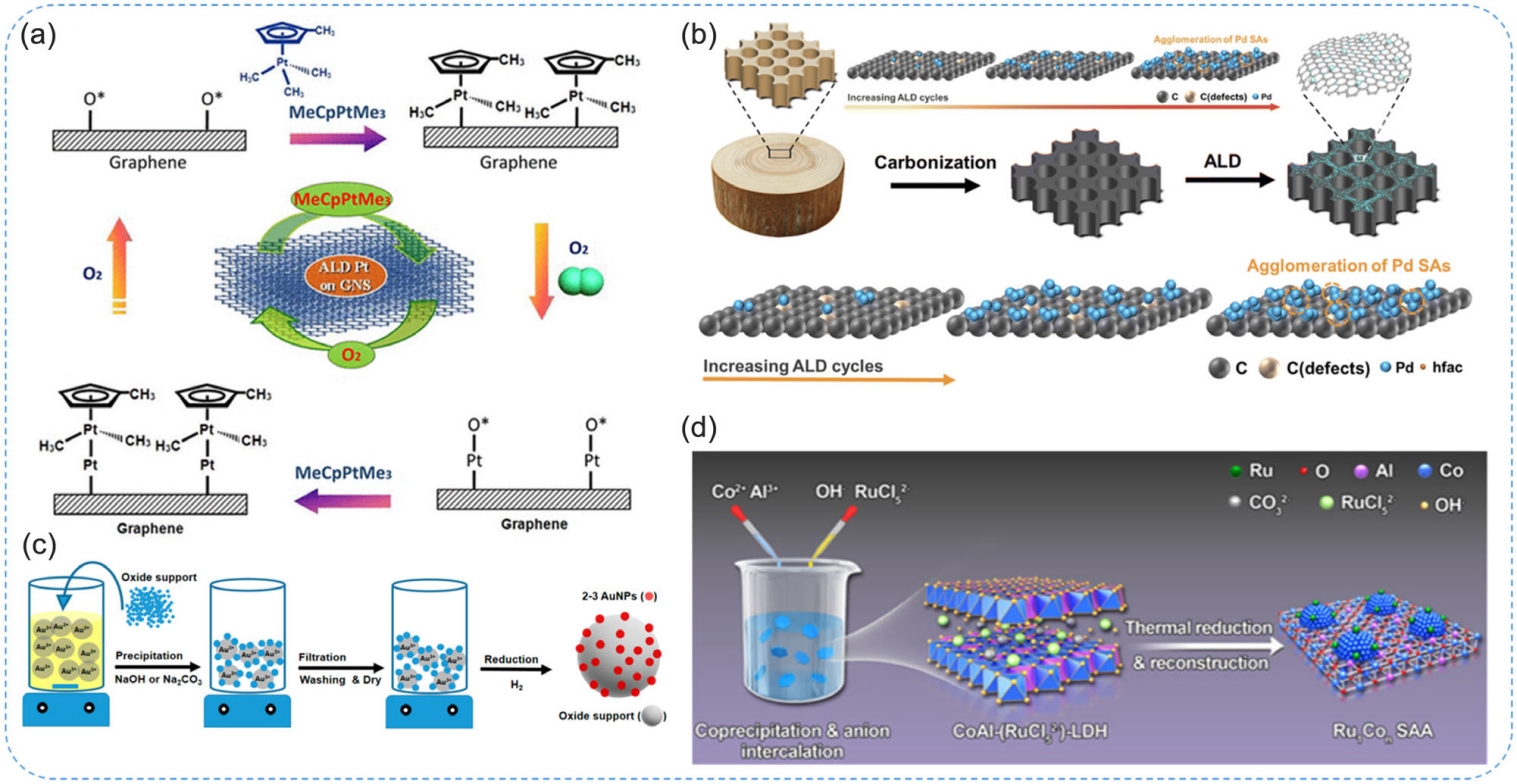
ALD technique and deposition‐precipitation method for SAC synthesis. a) Pt SA synthesized on graphene nanosheets via a layer‐by‐layer thin film deposition mechanism. Reproduced with permission from ref. [62] Copyright 2013, Springer Nature. b) Illustrative diagram of the preparation of Pd SAs anchored on CW using ALD mechanism. Reproduced with permission from ref. [53] Copyright 2024, Royal Society of Chemistry. c) Illustration of deposition–precipitation method for the synthesis of SAC on supporting material. Reproduced with permission from ref. [57] Copyright 2019, MDPI. d) Schematic illustration of the strategy of anion intercalation and 2D confinement to synthesize Ru_1_Co_*n*_ SAA using deposition‐precipitation method. Reproduced with permission from ref. [64] Copyright 2021, American Chemical Society.

ALD is a highly controlled method for thin film deposition and enables the accurate placement of individual SAs on support materials, ensuring uniform distribution and minimizing aggregation, which is crucial for maximizing catalytic activity and stability.^[^ ^53^ ^]^ The inherent control provided by ALD facilitates the creation of SACs with well‐defined atomic sites, essential for achieving optimal catalytic performance in various catalytic reactions. Moreover, the ability to deposit multiple cycles with atomic precision enhances the reproducibility and scalability of SACs for industrial applications. Sun et al.^[^ ^62^ ^]^ described a practical method for synthesizing Pt SAs anchored to graphene nanosheets via ALD technique (**Figure** **7**a). Structural analyses revealed the presence of individual Pt atoms and extremely small clusters, suggesting a strong Pt‐C coordination motifs between graphene and Pt SAs. This interaction was further enhanced by the introduction of carbon vacancies in the graphene sheet, which strengthens the bonding between graphene and Pt_13_ clusters. In other reports, Niancai et al. Fe SACs were synthesized on various substrates, including multiwalled carbon nanotubes, SiO_2_, and TiO_2_, with high Fe loading (>1.5 wt%).^[^ ^63^ ^]^ Structural analyses confirmed that the isolated Fe atoms were stabilized through Fe–N coordination on carbon nanotubes and Fe–O coordination on oxide supports, providing strong anchoring sites that maintained atomic dispersion and enhanced catalytic activity. Additionally, Pd nanoparticles and Pd SAs were anchored on carbonized wood (CW) using ALD, where the isolated Pd atoms were stabilized through strong Pd‐O and Pd‐C coordination motifs provided by oxygenated and carbon defect sites in the CW support^[^ ^53^ ^]^ (Figure 7b). Advancements in ALD techniques for synthesizing high‐loading SACs are promising, as they enable precise control over the deposition process, which may facilitate commercialization and large‐scale applications. The capability of ALD to achieve uniform atomic dispersion on diverse supports not only enhances stability and catalytic performance but also effectively mitigates aggregation, thereby ensuring long‐term efficiency and reliability in practical catalytic systems.

#### 2.2.4 Deposition–Precipitation Method

Deposition‐precipitation is a technique in which metal ions react with an alkali to form metal hydroxides or carbonates that deposit onto the surface of a support, as illustrated in Figure 7c.^[^ ^57^ ^]^ This method enables uniform dispersion of SAs and offers control over metal loading and dispersion through precise adjustment of the metal salt concentration and pH of the solution.^[^ ^57^ ^]^ Furthermore, tailoring the anchoring sites on the support surface can enhance formation of strong M‐O, M‐N, or mixed coordination motifs, thereby improving catalyst stability. Meng et al.^[^ ^64^ ^]^ prepared a RuCo_*n*_ SACs via a deposition‐precipitation method as illustrated in Figure 7d, where isolated Ru atoms were stabilized through Ru–O–Co coordination motifs after thermal reduction. The final catalyst had a low Ru load of 0.2 wt%, which could restrict practical applications. Xiang et al.^[^ ^52^ ^]^ studied atomically dispersed Au catalysts with varying Au contents supported on cerium–zirconium solid supports, where Au–O and Au–O–Ce/Zr coordination motifs were confirmed as the key sites. DFT calculations combined with experimental results suggested that catalytic activity can be further tuned through synergistic interactions between neighboring Au SAs. Nishio et al.^[^ ^65^ ^]^ introduced an efficient method for depositing Au nanoparticles onto metal phosphates with aqueous ammonia. The formation mechanism of Au–O–P coordination motifs was facilitated by strong interactions between Au‐ammine complexes (derived from HAuCl_4_ and ammonia) and positively charged phosphate groups, leading to highly dispersed Au nanoparticles. These catalysts exhibited superior catalytic activity compared with those supported on simple metal oxides, particularly in the hydroamination of terminal alkynes. In summary, the deposition–precipitation method offers notable advantages, including simplicity, cost‐effectiveness, and suitability for synthesizing SACs with low metal loading. Strong coordination motifs such as M–O, M–N, and M–O–M play critical roles in anchoring isolated atoms and ensuring uniform dispersion. By precisely controlling solution chemistry and tailoring anchoring sites on supports, this approach enhances metal‐support interactions, stability, and catalytic performance. **Table** **1** succinctly compares top‐down and bottom‐up SACs synthesis approaches. Top‐down methods offer benefits like ease and control, but they face challenges in cost and uniformity. Bottom‐up methods provide simplicity and scalability but can suffer from stability and reproducibility issues. The key benefits of strategy on different synthesis methods impacted on sensor applications.

**Table 1:** Summary of synthesis method of SACs with their advantages, disadvantages and impact on sensor applications.

Approach	Synthesis methods	Advantages	Disadvantages	Synthesis impact on sensing	Ref.
Top‐down	Pyrolysis	Easy to prepare, cost‐effective	Easily agglomerate	Abundant M‐N motifs enhance catalytic activity and reproducibility	[30]
	Anti‐Ostwald ripening	Uniform particle size	Complex formulations	Prevents agglomeration, long‐term stability but scalability is limited	[31]
	High‐ temperature atom trapping	Strong atom substrate bonding Superior thermal stability	Costly equipment High energy consumption	Excellent durability in harsh environments; industrial feasibility is limited	[33]
	Mass‐selected soft landing	Controlled surface modification High specificity	Expensive method Low deposition efficiency	Useful for fundamental sensing studies to probe active sites; impractical for large‐scale devices	[41]
	Sacrificial template assisted	High surface area, uniform dispersion	High cost, template removal requires harsh condition	Enhance sensitivity, selectivity and continuous sensing reliability	[45]
Bottom‐up	Co‐precipitation	Simple and effective method Large‐scale production Size controllable	Reproducibility issue Limited metal loading	Enables low‐cost biosensors, but batch‐to‐batch variability reduces reliability	[50]
	Impregnation	Cost‐effective Large ‐scale production, high metal atom utilization	Weaker metal ‐support interactions Aggregation	Widely applied in electrochemical sensors; stability is a key challenge under real conditions	[51]
	Deposition‐precipitation	Well‐dispersed deposition Strong metal‐support interaction Low‐cost method	Stability issue Sensitive to reaction conditions	Good for selective sensing applications; requires fine control for reproducible detection	[52]
	ALD	Control thickness Uniform dispersion Reproducibility Stability	Slow deposition rate, expensive method	Produces highly stable sensors with atomic precision; but cost hinders large‐scale adoption	[53]

## 3 Characterization Techniques of SACs

**Figure 8:**
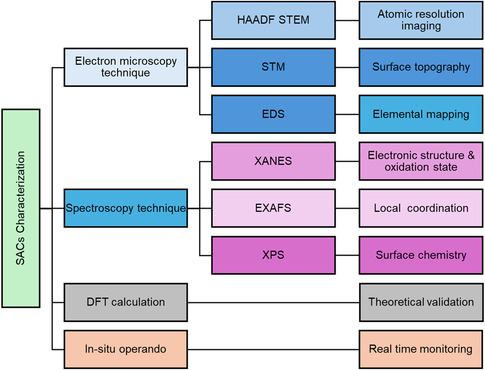
Schematic representation of various advanced characterization techniques commonly employed for the analysis of SACs, including structural identification, electronic configuration, surface chemistry, and local coordination environments.

Identifying the presence and electronic state of isolated SAs on a support poses significant analytical challenges but is essential for the development of SACs. Owing to their atomic‐scale dispersion and strong interaction with the surrounding matrix, SACs demand the use of advanced and often complementary characterization techniques to unravel their structural, electronic, and catalytic properties.^[^ ^66^ ^]^ These techniques can be broadly categorized into three main groups: electron microscopy, spectroscopic methods, and DFT calculations. Each of these techniques offers complementary insights into the formation mechanisms, structural and catalytic properties of SACs as shown in **Figure** **8**.

### 3.1 Electron Microscopy Characterization

**Figure 9:**
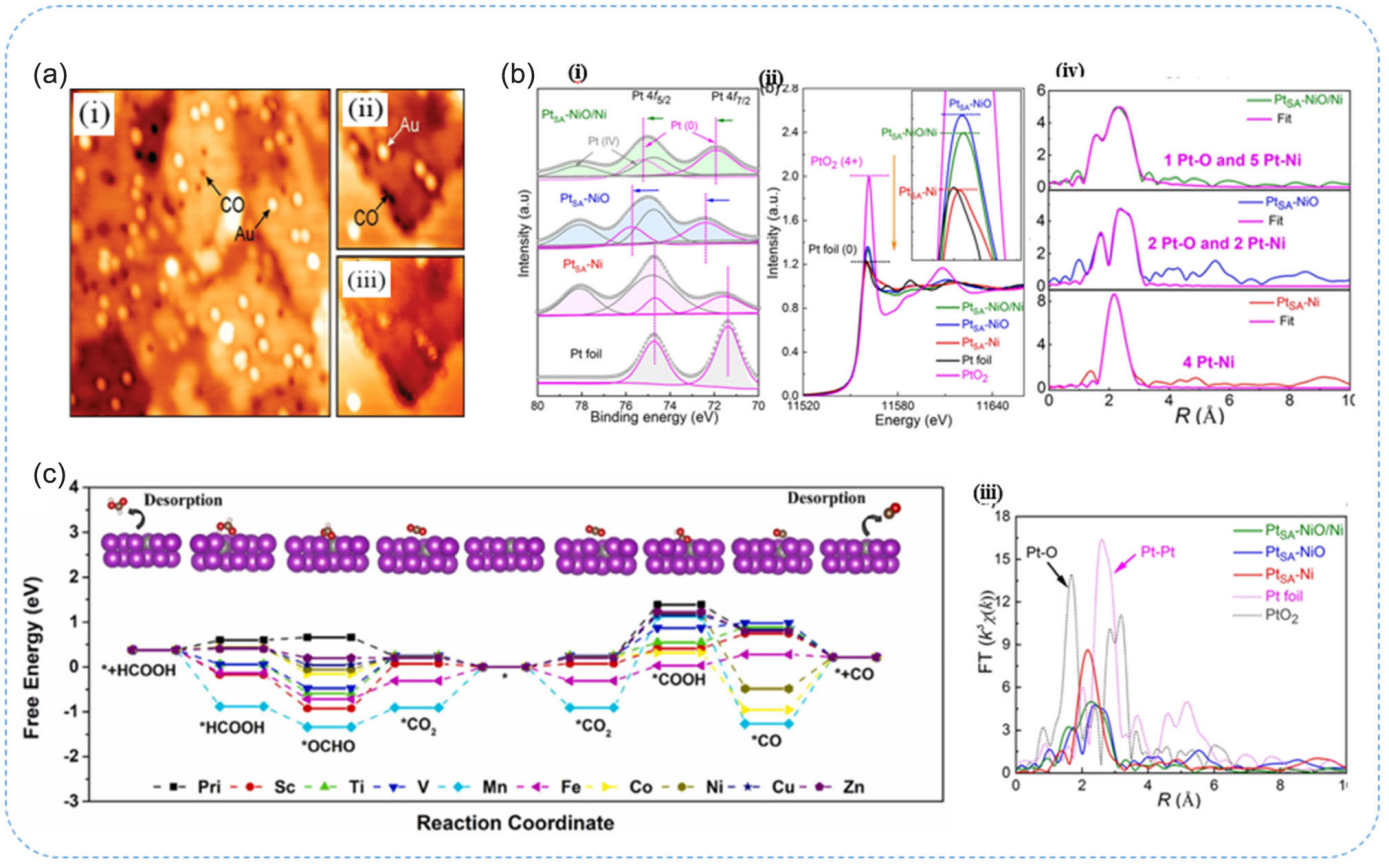
a) STM images of isolated Au atoms and CO molecules on MgO thin films (i–iii). Reproduced from ref. [68] Copyright 2010, American Chemical Society. b) Spectroscopic studies of Pt SA‐NiO/Ni (i) XPS of Pt 4*f* spectra, (ii) XANES spectra, (iii) corresponding FT‐EXAFS curves, and (iv) EXAFS fitting curve of Pt_SA_‐NiO/Ni, Pt_SA_‐NiO, and Pt_SA_‐Ni *R*‐space. Reproduced with permission from ref. [73] Copyright 2021, Springer Nature. c) Theoretical free‐energy landscape for CO_2_ reduction over Bi and SAA catalyst surfaces, highlighting differences in reaction pathways and energy barriers. Reproduced with permission from ref. [70] Copyright 2023, Elsevier.

Electron microscopy techniques reveal the local atomic arrangement, enabling the study of the coordination environment of SAs within the catalyst matrix. At the atomic level, techniques such as transmission electron microscopy (TEM), scanning transmission electron microscopy (STEM), and HAADF‐STEM offer real‐space images, allowing for the direct visualization of SA dispersion and the overall structure of the catalyst. Its Z‐contrast imaging allows direct identification of individual heavy atoms anchored on lighter supports and even the local atomic coordination environment can be inferred. For example, Qin et al.^[^ ^67^ ^]^ investigated Pt SACs dispersed on anatase TiO_2_ thin films at varying densities to evaluate their photocatalytic performance under different light intensities. HAADF‐STEM was used to examine the distribution of Pt atoms deposited from precursor solutions of different concentrations. The imaging revealed atomically dispersed Pt species anchored on the TiO_2_ surface, with a characteristic lattice spacing of 0.354 nm corresponding to the (101) crystal plane of anatase TiO_2_. High‐resolution STEM further confirmed the presence of a well‐defined TiO_2_ lattice structure, validating the structural integrity of support. Quantitative analysis of multiple HAADF‐STEM images revealed Pt coverages of *θ* = 0.29% for 0.005 mM H_2_PtCl_6_, *θ* = 0.58% for 0.05 mM H_2_PtCl_6_, and *θ* = 2.12% for 2 mM H_2_PtCl_6_, demonstrating a clear correlation between precursor concentration and SA loading. Additionally, STM was employed to probe the geometric and electronic structures of the SACs, enabling identification of specific active sites and their interactions with adsorbed molecules, as illustrated in **Figure** **9**a.^[^ ^68^ ^]^ Yang et al.^[^ ^69^ ^]^ synthesized Pd‐Cu SA alloys by evaporating a trace amount of Pd adatoms (≈0.01 monolayer) onto a Cu (111) substrate while maintaining the substrate temperature at 380 K. The corresponding STM image displays significantly brighter chemical contrasts for the Pd atoms compared to the host Cu atoms, confirming the successful atomic‐scale incorporation of Pd into the Cu lattice. Along with STEM imaging, the EDX technique was employed to predict the uniform dispersion of elements, detect SA sites, and identify local compositional variations in heterogeneous catalysts. In contrast, non‐atomic‐level electron microscopy techniques, conventional TEM and field‐emission scanning electron microscopy (FE‐SEM) are effective in examining morphology, particle size, and surface features, but lack the resolution to unambiguously identify isolated atoms. Shen et al.^[^ ^70^ ^]^ synthesized the SAA‐ZnBi catalyst using a two‐step in‐situ electrochemical reduction strategy. FE‐SEM revealed that the nanosheet‐like morphology of the Zn‐Bi_2_S_3_ precursor was well retained after the synthesis. Additionally, EDX elemental mapping demonstrated the uniform distribution of Zn atoms within the Bi nanosheets. Further, HAADF‐STEM revealed the atomic dispersion of Zn atoms in the Bi matrix, providing preliminary confirmation of the successful formation of the Zn SAA structure.

Electron microscopy techniques offer powerful direct imaging capabilities and valuable insights into the structural and electronic properties of SACs and their supports; they are limited to providing only localized surface information. These techniques may fail to detect SAs embedded within the bulk of the catalyst. Therefore, complementary characterization methods like in situ operando and spectroscopic studies are necessary to fully validate the distribution and location of SAs within catalytic systems.

### 3.2 Spectroscopy Studies

Spectroscopic methods enable both bulk and surface‐sensitive characterization, providing essential information to confirm the oxidation state, dispersion, and coordination geometry of SAs, even when they are embedded within the catalyst matrix. At the non‐atomic methods such as X‐ray photoelectron spectroscopy (XPS), Mössbauer spectroscopy, and infrared (IR) spectroscopy provide valuable insights into electronic structure, oxidation states, and ligand interactions at the molecular level. In contrast, atomic level techniques such as XAS, XANES, and EXAFS directly reveal the oxidation state, electronic environment, and coordination of isolated atoms which offer localized imaging.^[^ ^71^, ^72^ ^]^ Zhou et al.^[^ ^73^ ^]^ introduced a highly efficient Pt SACs immobilized on a NiO/Ni heterostructure. Pt SA, coupled with the NiO/Ni heterostructure, tunes the binding energies of hydroxyl ions (OH*) and hydrogen (H*). Further performance enhancement is achieved by constructing Pt SA‐NiO/Ni nanosheets on Ag nanowires to form a hierarchical 3D morphology, which was observed in electron microscopic studies such as FE‐SEM, AC‐HAADF STEM, and EDX. Further, the author studied Pt SA‐NiO/Ni electronic and coordination geometry via XANES, EXAFS, and XPS, as shown in Figure 9b. In XPS analysis, Pt 4f spectra revealed Pt species in all catalysts are close to Pt^0^ but exhibit varying degrees of positive shifts compared to Pt foil, indicating electrochemical reduction of PtCl_6_^2−^ and charge transfer from the Pt sites to the supports (Figure 9b(i)). Notably, Pt SA‐NiO displays the largest positive shift in its Pt 4f spectrum, suggesting the highest electron loss of Pt. Additionally, Pt XPS fitting reveals the presence of Pt (IV) species, attributed to adsorbed PtCl_6_^2−^ ions. XAFS measurements further confirm the electronic environment of Pt, with Pt *L*_3_‐edge. As shown in Figure 9b(ii), XANES spectra shows distinct changes in white‐line peak intensity across different supports. The decrease in white‐line intensity from NiO to NiO/Ni to metallic Ni, reflects an increase in the 5d occupancy of Pt, indicating less charge loss in Pt when coordinated with more metallic supports. However, the differential XANES spectra (Figure 9b(iii)) revealed the average valence state of Pt increases from +0.29 (Pt SA‐Ni) to +1.23 (Pt SA‐NiO), indicating a higher oxidation state of Pt with stronger interactions with the support. In addition (Figure 9b(iv)), EXAFS analysis shows no Pt–Pt bonding in the catalysts, confirming the dispersion of Pt SAs. The coordination environment of Pt in Pt SA‐NiO/Ni includes one O and five Ni atoms, while in Pt SA‐NiO, Pt is coordinated with two O and two Ni atoms, and in Pt SA‐Ni, Pt is coordinated with five Ni atoms. Wavelet transform analysis further confirms the interfacial coordination of Pt in NiO/Ni with no Pt–Pt coordination detected, supporting the successful dispersion of Pt SAs. These findings highlight the electronic interactions between Pt and NiO/Ni, providing insights into optimizing Pt‐based catalysts for enhanced performance.

Overall XANES helps determine the oxidation state and electronic environment of metal atoms, while EXAFS reveals the local coordination and bonding of the metal to its support. XPS provides detailed insights into the chemical states and electronic interactions between the metal atoms and their support. Altogether these techniques enable the design and optimization of SACs by offering a deeper understanding of their structure‐activity relationships.

### 3.3 Density Functional Theory

DFT calculations serve as a powerful computational approach for investigating the electronic structure and catalytic reactivity of SACs. These simulations offer fundamental insights into the interaction between SACs and their supporting materials, as well as the underlying mechanisms of catalytic processes.^[^ ^74^ ^]^ By modeling the atomic and electronic configurations of SAC systems, DFT enables the prediction of catalytic activity, stability, and selectivity. Shen et al.^[^ ^70^ ^]^ investigated a Zn SA alloyed Bi catalyst (SAA‐ZnBi) for electrocatalytic CO_2_‐to‐formate conversion using DFT calculations. By modeling the atomic structure and simulating the adsorption behavior of key intermediates, particularly *OCHO, the calculations revealed that introducing isolated Zn SAs into the Bi matrix effectively tunes the electronic environment of the catalyst (Figure 9c). This modification results in a balanced adsorption strength of *OCHO, which is crucial for promoting efficient CO_2_‐to‐formate conversion while preventing catalyst poisoning or desorption. The reaction energy profiles indicated that *OCHO formation is a potential‐determining step and the energy barrier for this process is notably reduced on SAA‐ZnBi compared with pure Bi. Additionally, electronic structure analysis, including charge distribution and density of states further confirmed that Zn atoms function as electron donors, enhancing the interaction with CO_2_ intermediates. These theoretical insights guided the synthesis of SAA‐ZnBi and aligned with the superior catalytic performance observed experimentally.

Overall, DFT studies enable the prediction of reaction energetics, optimization of metal‐support interactions, and the design of catalysts with tailored properties for specific reactions. Additionally, DFT analysis evaluates the stability and durability of SACs under operational conditions, providing valuable insights to guide experimental design and enhance catalytic performance.

### 3.4 In Situ Operando Technique

In situ characterization techniques such as XAS, Raman spectroscopy, UV‐visible spectroscopy, and attenuated total reflectance surface‐enhanced infrared absorption spectroscopy (ATR‐SEIRAS) play a critical role in enabling real‐time monitoring of structural transformations, metal‐support interactions, and the dynamic evolution of active sites. These techniques provide essential insights into reaction mechanisms, catalyst stability, and deactivation pathways, thereby informing the rational design and optimization of catalytic systems.^[^ ^75^ ^]^ Ding et al. investigated the dynamic behavior and degradation mechanisms of metal‐nitrogen‐carbon (M‐N‐C) SACs, focusing specifically on Cu‐N‐C systems. Utilizing a combination of in situ spectroscopic techniques, including ATR‐SEIRAS and XAS, they successfully tracked surface‐bound intermediates and monitored changes in the oxidation state and coordination environment of the Cu active sites, respectively. Additionally, quasi‐in situ electron paramagnetic resonance (EPR) and in situ UV‐vis spectroscopy confirmed the generation of hydrogen radicals, which actively cleave Cu—N bonds, leading to Cu^2+^ leaching and subsequent reduction into Cu nanoparticles. These results showed that Cu_1_/N_pyrrolic_‐C has a Cu nanoparticle formation rate over six times higher than Cu_1_/N_pyridinic_‐C, highlighting the crucial role of coordination environment in stability.^[^ ^76^ ^]^ Chen et al.^[^ ^77^ ^]^ designed UiO‐67 MOF embedded with N‐heterocyclic carbene (NHC) coordinated Cu SACs for the electrosynthesis of methane (CH_4_) under ambient condition. The structural and catalytic properties of the UiO‐67 MOF/NHC‐Cu SACs were elucidated using in situ Raman spectroscopy and ATR‐SEIRAS. These analyses confirmed that Cu SACs incorporated NHC maintain stable Cu species during the CO_2_ reduction reaction, preserving their oxidation state and coordination structure. Moreover, key reaction intermediates such as COOH* and OCH_3_* were clearly detected, with significantly stronger signals observed on the UiO‐67 MOF/NHC–Cu catalyst, indicating improved intermediate stabilization and enhanced CH_4_ production efficiency. Together, these studies highlight the indispensable role of in situ characterization techniques in uncovering the stability, degradation pathways, and mechanistic details of SACs. They also emphasize how tuning the coordination environment, whether through nitrogen species or ligand functionalization, can significantly impact catalytic performance, guiding the rational design of more durable and efficient single‐atom catalysts.

## 4 Supporting Materials for Single Atom Catalysts (SACs)

When the dimensions of nanocatalysts are reduced to the atomic scale, metal atoms with elevated surface energy become prevalent. As a result, isolated SAs exhibit a strong tendency to aggregate. To moderate this, various nanomaterials—such as MOFs, carbon‐based materials, and MO_*x*_—have been widely employed as effective supports to anchor individual metal atoms.^[^ ^16^, ^78^, ^79^, ^80^, ^81^, ^82^ ^]^ Specifically, interactions between the SAs and the support material confer stability, enabling a high surface concentration of active sites. Notably, the coordination environment of SACs with supporting materials plays a critical role in determining their catalytic efficiency. By tuning the coordination number and tailoring the support material, SACs can be optimized to accommodate a broad range of catalytic reactions.^[^ ^83^, ^84^, ^85^ ^]^ In general, the selection of catalyst support for sensor applications is a complex task that requires careful consideration of multiple factors, including the support material, target analyte, and sensor type.^[^ ^86^, ^87^, ^88^, ^89^ ^]^ Consequently, achieving optimal performance across different sensor platforms may necessitate the use of distinct SAC systems, each based on support materials specifically engineered for detection requirements.

### 4.1 SACs Based on Carbon Materials

**Figure 10:**
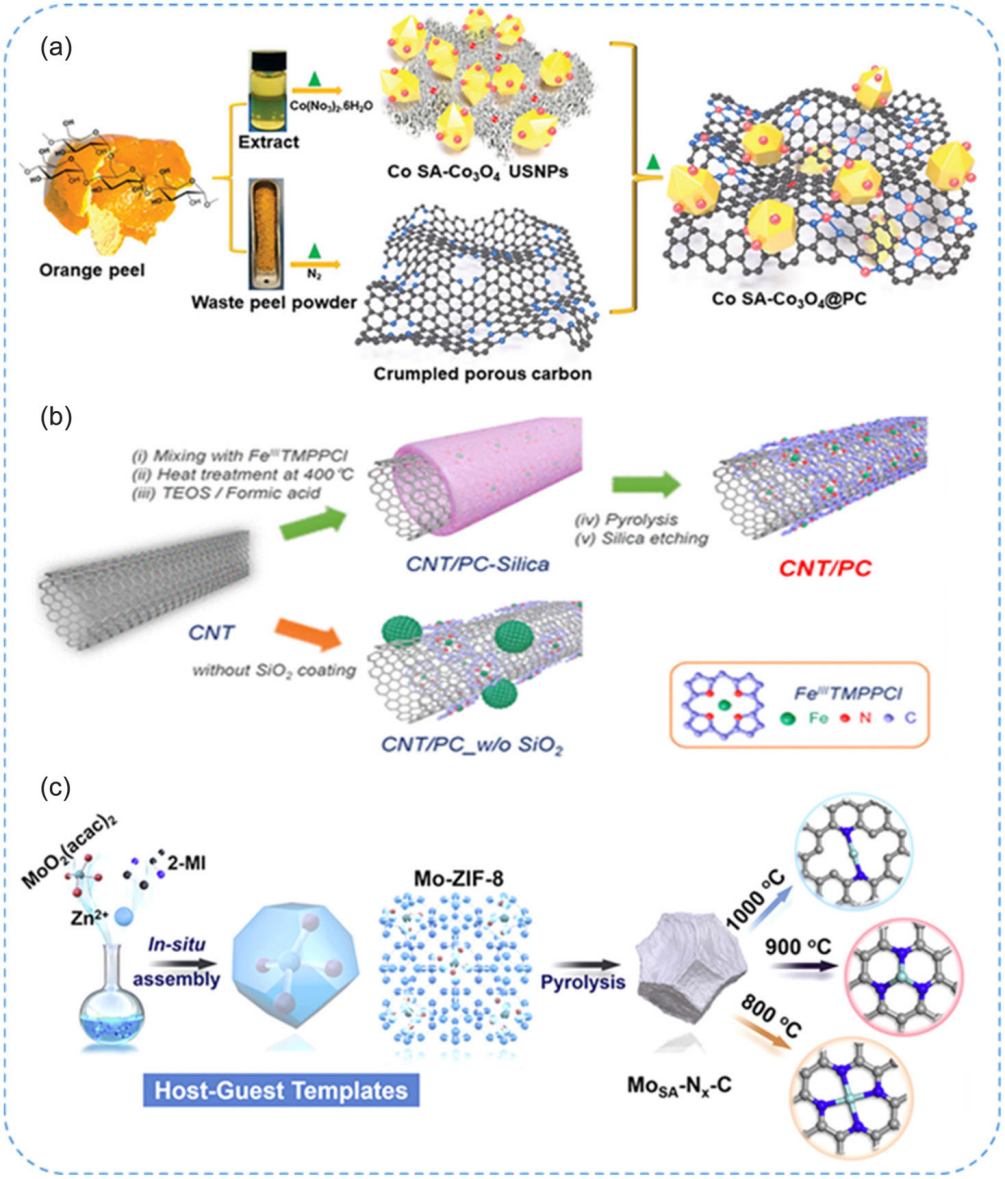
a) Schematic diagram for the synthesis of CoSA‐Co_3_O_4_@PC. Reproduced with permission from ref. [94] Copyright 2024, Wiley. b) Synthetic scheme for the preparation of Fe‐CNT/PC catalysts. Reproduced with permission from ref. [95]. Copyright 2016, American Chemical Society. c) Schematic illustration for the fabrication strategy of Mo–N_*x*_–C catalysts. Reproduced with permission from ref. [96] Copyright 2021, Elsevier.

Carbon‐based materials have emerged as one of the most widely used supports for SACs due to their exceptional physicochemical properties. Materials such as graphene, CNTs, carbon black, activated carbon, carbon fibers, and porous carbon frameworks offer high surface area, excellent electrical conductivity, chemical stability, and tunable surface functionalities.^[^ ^8^, ^90^, ^91^, ^92^ ^]^ These features make carbon materials highly effective in stabilizing isolated metal atoms and facilitating efficient charge transfer during catalytic reactions. The abundant defect sites, heteroatom doping (i.e., nitrogen, sulfur, phosphorus), and functional groups on carbon surfaces provide strong anchoring sites that prevent the aggregation of SAs. Additionally, the conductive nature of carbon support enhances electron transport, which is crucial for electrocatalytic and sensing applications. By tailoring the microstructure, pore size, and surface chemistry of carbon materials, the dispersion, coordination environment, and electronic properties of the SAs can be precisely controlled, leading to improved catalytic activity, selectivity, and stability.^[^ ^8^ ^]^ For instance, Wan et al.^[^ ^93^ ^]^ synthesized a biomass‐inspired coordination confinement strategy to fabricate Fe, Co, and Ni SACs. Using in situ XAS, STEM, and EPR analyses revealed that SAs dynamically rearrange into amorphous (oxy)hydroxide clusters, where oxygen‐bridged dual‐metal moieties (M–O–M/M′) serve as the true active sites. Venkateswarlu et al.^[^ ^94^ ^]^ prepared Co SACs embedded in Co_3_O_4_ nanoparticles and porous carbon (CoSA‐Co_3_O_4_@PC) using waste orange peel as the sole feedstock (**Figure** **10**a). The EXAFS analysis revealed that Co SA sites were coordinated as Co‐Co_3_O_4_ and Co‐N_4_ on PC. Theoretical studies indicate that optimized Co SA sites lower the energy barrier for water dissociation and enhance the performance via the adsorbate evolution mechanism. Joo et al.^[^ ^95^ ^]^ developed a universal “silica‐protective‐layer‐assisted” strategy to selectively form monodispersed FeN_*x*_ active sites while minimizing the formation of less‐active large Fe particles. In this approach, a porphyrinic precursor mixed with CNTs was coated with a silica layer, followed by high‐temperature pyrolysis and silica etching to yield the Fe‐N/C catalyst (Figure 10b). Cui et al.^[^ ^96^ ^]^ demonstrated that Mo‐N_*x*_‐C SACs exhibit targeted peroxidase‐like activity with selectivity governed by the coordination number of Mo sites. Among Mo‐N_2_‐C, Mo‐N_3_‐C, and Mo‐N_4_‐C, Mo‐N_3_‐C catalyst showed the highest peroxidase‐like activity and the lowest oxidase‐like activity. These findings highlight the strong structure–selectivity relationship, offering a strategy for rationalizing highly specific nanozymes (Figure 10c). Duan et al.^[^ ^97^ ^]^ decorated monodispersed atomic metals (Fe, Co, Ni) into N‐doped graphene (NG) lattices with an MN_4_C_4_ structure using a two‐step synthesis strategy, revealing the synthesis–structure–property relationship. Nitrogen dopants in the graphene framework were introduced either by adding a nitrogen source or by selecting nitrogen‐containing precursors. Chen et al.^[^ ^98^ ^]^ demonstrated a novel sulfur‐assisted strategy that was developed to achieve atomic dispersion of Fe‐N_*x*_ species on nitrogen and sulfur co‐decorated hierarchical carbon layers, producing SACs. The abundant Fe‐N_*x*_ active sites, improved electrical conductivity, and hierarchical structure enhance active site exposure and accelerate electron transfer, resulting in superior catalytic performance. In summary, carbon‐based materials are highly effective supports for SACs due to their high surface area, conductivity, and ability to form strong coordination motifs. Heteroatom doping, defect engineering, and hierarchical structuring enable stable atomic dispersion and tunable electronic properties, leading to enhanced catalytic activity and selectivity.

### 4.2 SACs Based on Metal Oxides

**Figure 11:**
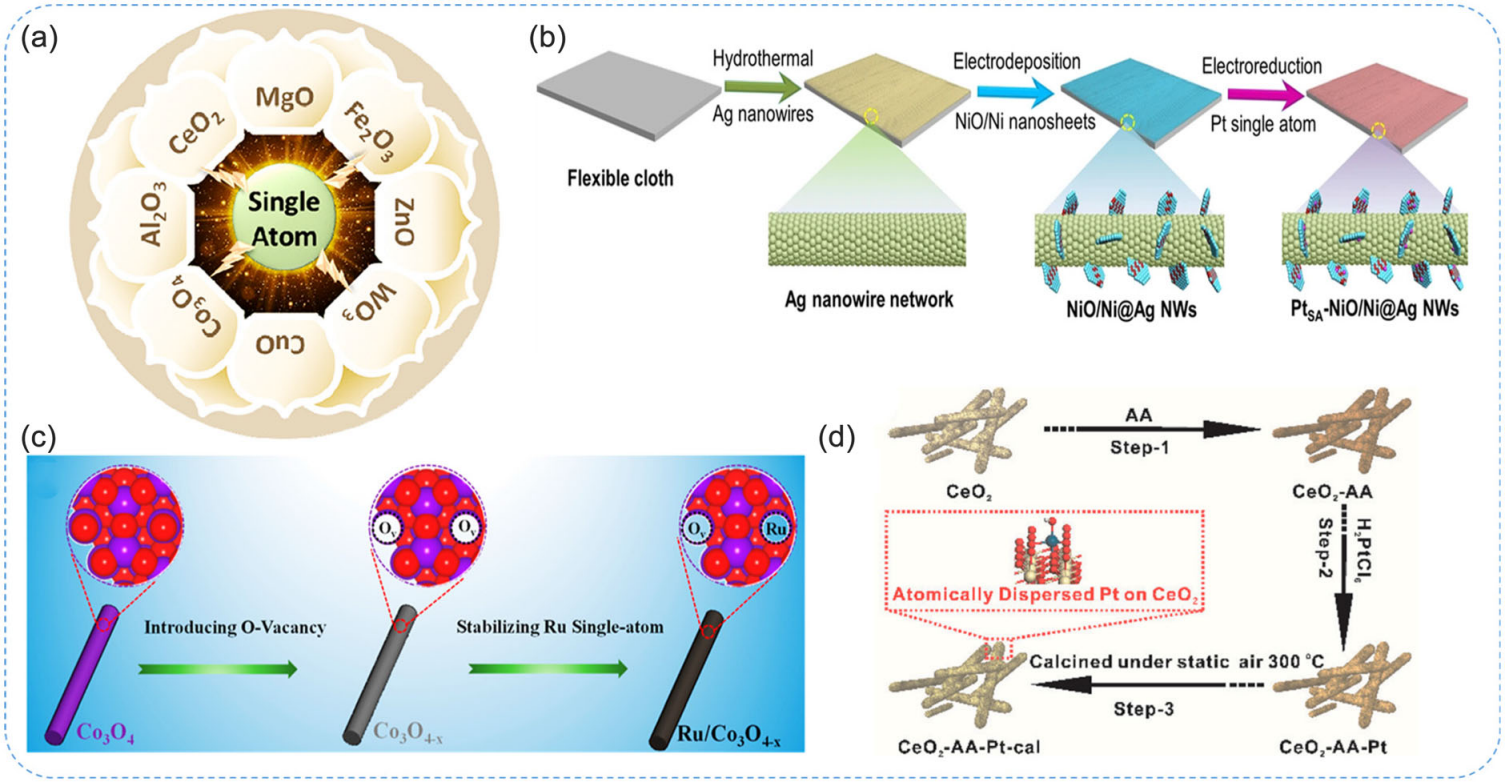
a) Schematic illustration of SACs with metal oxides. Reproduced from ref. [16] Copyright 2020, American Chemical Society. b) The synthesis process of Pt SA anchored NiO/Ni heterostructure nanosheets on Ag nanowires network. Reproduced with permission from ref. [73] Copyright 2021, Springer Nature. c) Illustration of preparing Ru/Co_3_O_4–*x*_ electrocatalyst via the defect‐induced in situ synthesis strategy. Reproduced from ref. [100] Copyright 2023, American Chemical Society. d) Schematic illustration of the l‐ascorbic acid (AA)‐assisted reduction synthesis of the atomically dispersed Pt/CeO_2_ catalyst (CeO_2_‐AA‐Pt). Reproduced with permission from ref. [101] Copyright 2018, American Chemical Society.

Among various supports for SACs, oxide‐based materials (e.g., TiO_2_, FeO_*x*_, NiO, ZnO, Al_2_O_3_, CeO_2_, etc.,) have attracted particular interest due to their abundant defect sites (steps, corners, vacancies) and surface ‐OH groups, which serve as anchoring points for SAs (**Figure** **11**a).^[^ ^16^, ^82^, ^99^ ^]^ The strong interactions between MO_*x*_ and SAs enhance catalytic performance through synergistic effects. Additionally, the high thermal stability of MO_*x*_ contributes significantly to the mechanical and thermal robustness of SACs. Furthermore, the single metal atoms can substitute surface cations on MO_*x*_ and strongly anchor through interactions with nearby oxygen anions.^[^ ^16^, ^81^ ^]^ For example, Zhou et al.^[^ ^73^ ^]^ developed a 3D nanostructured catalyst composed of 2D NiO/Ni heterostructure nanosheets with Pt SA anchored onto a 1D Ag nanowire (Ag NW). The Ag NWs were first synthesized via a hydrothermal method and loaded onto flexible cloth (≈0.47 mg cm^−2^), forming a uniform, porous conductive network. The Ni/NiO composite was then attached to the Ag NW network through a straightforward electrodeposition process (Figure 11b). DFT calculations reveal that dual active sites‐metallic Ni and O‐vacancy‐modified NiO at the NiO/Ni interface improve catalytic performances. Yuan et al.^[^ ^100^ ^]^ fabricated atomically dispersed Ru supported on oxygen‐deficient Co_3_O_4‐*x*_ (Ru/Co_3_O_4‐*x*_) by using a simple impregnation method (Figure 11c). Co_3_O_4‐*x*_ was first prepared by immersing Co_3_O_4_ into NaBH_4_ solution under ultrasonic vibration, followed by washing and drying. Subsequently, RuCl_3_ aqueous solution was added to the Co_3_O_4‐*x*_ dispersion, stirred for 2 h and then centrifuged and dried, to obtain Ru/Co_3_O_4‐*x*_. DFT calculations revealed that oxygen vacancies and Ru atoms synergistically modulate the electron distribution and d‐band center of Co_3_O_4_, optimizing oxygen intermediate adsorption and lowering reaction barriers. This defect‐induced in situ SA deposition strategy offers a promising approach for designing advanced catalysts performance. Recently, Chen et al.^[^ ^101^ ^]^ stabilized atomically dispersed Rh sites on CeO_2_ supports via an impregnation method. The CeO_2_‐based support was prepared using cerium (III) nitrate, zirconium (IV) oxynitrate, and ammonium hydroxide, followed by Rh deposition using rhodium (III) acetylacetonate in ethanol. After drying and calcination, an Rh/CeO_2_ catalyst with 0.4 wt% Rh loading was obtained (Figure 11d). The doped CeO_2_ support efficiently trapped Rh atoms, preventing agglomeration. Correspondingly, CeO_2_ has been employed to support Pt and Pd SAs, benefiting from its redox activity and distinct acid‐base bifunctionality. These properties critically influence both surface adsorption characteristics and the behavior of the supported metal species. Koga et al.^[^ ^102^ ^]^ demonstrated that atomically dispersed Pd additives significantly enhance the hydrogen sensing performance of Co_3_O_4_ nanoparticle films. Pd was introduced via pulsed laser ablation, forming SAs at low concentrations (5% Pd loading) and oxide clusters at higher concentrations. Single Pd atoms (Pd^4+^) substituted Co^3+^ sites, donating electrons to Co_3_O_4_ and increasing ionosorbed oxygen, thus boosting sensitivity. A reversible Pd^4+^ ↔ Pd^2+^ catalytic redox cycle further accelerated hydrogen reactions, providing deep insights into the role of noble metal SAs in gas sensing. Xu et al.^[^ ^103^ ^]^ investigated Ir SAs anchored on WO_3_ supports (Ir_1_–WO_3_), developing a strong metal–support interaction (EMSI). XANES spectra at the Ir L_3_‐edge confirmed atomic dispersion and strong electronic interaction between Ir and WO_3_. DFT calculations showed that EMSI caused significant electron redistribution, lowering the energy barrier for the ring‐opening step. This study highlights the crucial role of EMSI in boosting the catalytic activity, stability, and industrial relevance of SACs. Recently, Hong et al.^[^ ^104^ ^]^ developed a 2D‐nanoconfined atomic‐site grafting strategy to synthesize high‐density SACs on porous MO_*x*_ nanosheets. Using a bilayer silica envelope, single transition metal hydroxide layers with metal precursors were thermally converted into uniformly dispersed single metal atoms on 1‐nm‐thin MO_*x*_ nanosheets, preventing aggregation. This versatile method enabled the grafting of various metals (e.g., Pt, Ir) and dual metal sites on 2D‐NiO supports, offering a scalable route for high‐performance SACs. Overall, MO_*x*_ are highly effective supports for SACs due to their abundant defect sites, oxygen vacancies, and strong M‐O coordination motifs that stabilize isolated atoms. These features not only prevent aggregation but also tune electronic structures through strong metal‐support interactions, leading to enhanced activity and durability. Such properties make oxide‐supported SACs promising candidates for electrocatalysis, gas sensing, and other industrially relevant applications

### 4.3 SACs Based on MOFs

**Figure 12:**
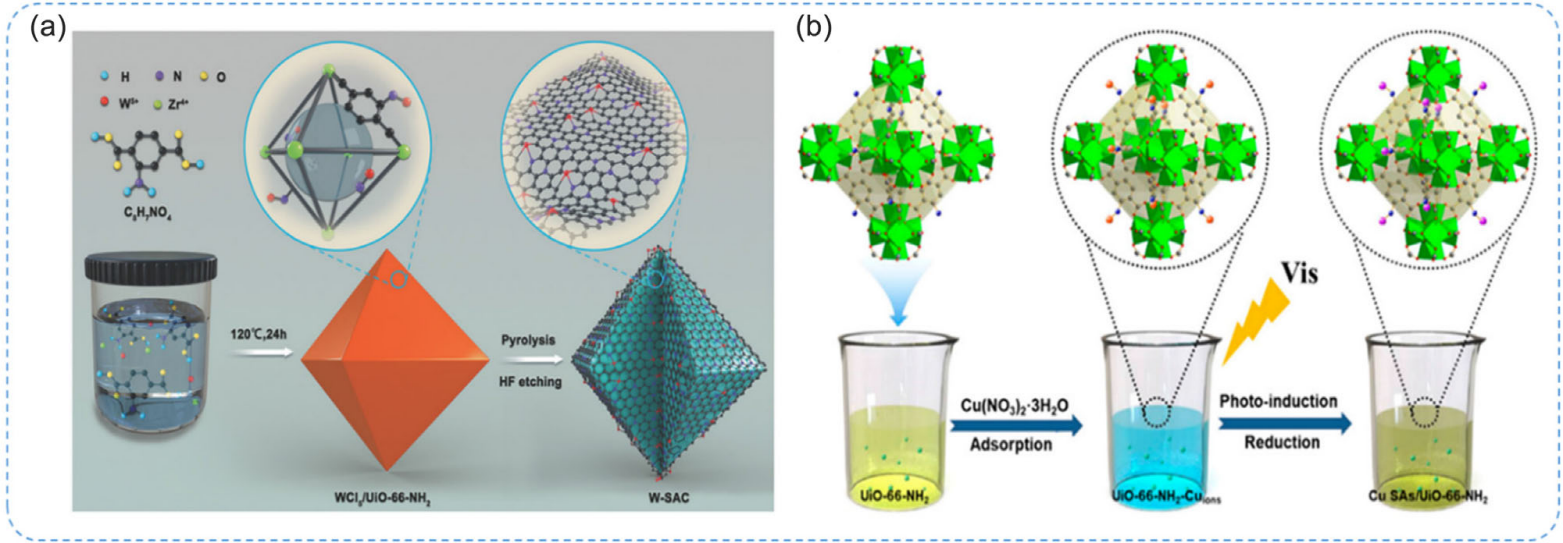
a) Synthesis of MOF‐derived W SACs through the pyrolysis approach. Reproduced with permission from ref. [106] Copyright 2018, Wiley. b) Synthesis process of the Cu SAs/UiO‐66‐NH_2_ catalyst using a ligand‐stabilizing strategy. Reproduced with permission from ref. [107] Copyright 2020, American Chemical Society.

MOFs are porous materials featuring well‐defined channels, high surface areas, diverse structures, and easy molecular‐level functionalization. Built from metal nodes and multifunctional organic ligands, MOFs provide abundant anchoring sites, enabling uniform dispersion of SAs through coordination, adsorption, or ion exchange.^[^ ^10^, ^99^ ^]^ Thus, MOFs serve as highly effective and versatile platforms for developing SACs with high metal loadings and excellent stability. Additionally, MOF‐derived materials‐particularly N‐doped porous carbons produced by pyrolysis can also be used, as they retain key structural features and properties of the original MOFs. For instance, Gayathri et al.^[^ ^105^ ^]^ synthesized MXene–Fe‐MOF/Ni SACs composites through sonochemical and pyrolysis methods. Morphological and analytical studies confirmed the hybrid structure of MXene and Fe‐MOF with Ni SAs coordinated to MXene via Ni–C bonds. The immobilization of Ni SACs at MXene vacancy sites significantly enhanced the catalytic performance of the hybrid material. Chen et al.^[^ ^106^ ^]^ reported a W SACs supported on MOF‐derived N‐doped carbon (NC) for efficient and stable catalytic performance (**Figure** **12**a). HAADF‐STEM and XAFS confirmed atomic dispersion of W species, identifying the W_1_N_1_C_3_ moiety as the likely active structure. DFT calculations showed that this unique structure enhances catalytic performance, offering new insights into W‐based catalyst design. Wang et al.^[^ ^107^ ^]^ developed Cu SACs using a ligand‐stabilizing strategy, where Cu precursors are captured by ‐NH_2_ groups in UiO‐66‐NH_2_ MOFs (Figure 12b). A mild photo‐induced method preserved the MOF's porous structure, forming a two‐coordinated Cu‐N planar structure that stabilized Cu atoms. This modification turned the semiconductor's electronic structure, lowering the conduction band energy and narrowing the band gap to enhance photoelectron enrichment. Park et al.^[^ ^108^ ^]^ synthesized SAC‐functionalized conductive MOFs (cMOFs) via cathodic electrochemical deposition using Pd, Ag, and Ir precursors. XAFS and DFT analyses showed that SACs are stabilized between 1D pore walls, coordinated with N atoms. The functionalized cMOFs retained their pore volume and surface area, preserving gas diffusion. Especially, Pd SACs particularly enhanced NO_2_ sensing by providing stable binding sites, reversible charge transfer, and structural stability, leading to improved sensitivity and durability compared with nanoparticle‐functionalized cMOFs. In summary, MOFs and MOF‐derived materials are excellent supports for SACs due to their tunable porosity, abundant anchoring sites, and precise molecular functionalization. Coordination motifs such as M‐N, M‐C, and M‐N‐C enable uniform atomic dispersion and improved stability, while also tuning the electronic structure for enhanced performance. These properties make MOFs highly promising platforms for designing advanced SACs in catalysis and sensing applications.

### 4.4 SACs Based on High Entropy Materials

**Figure 13:**
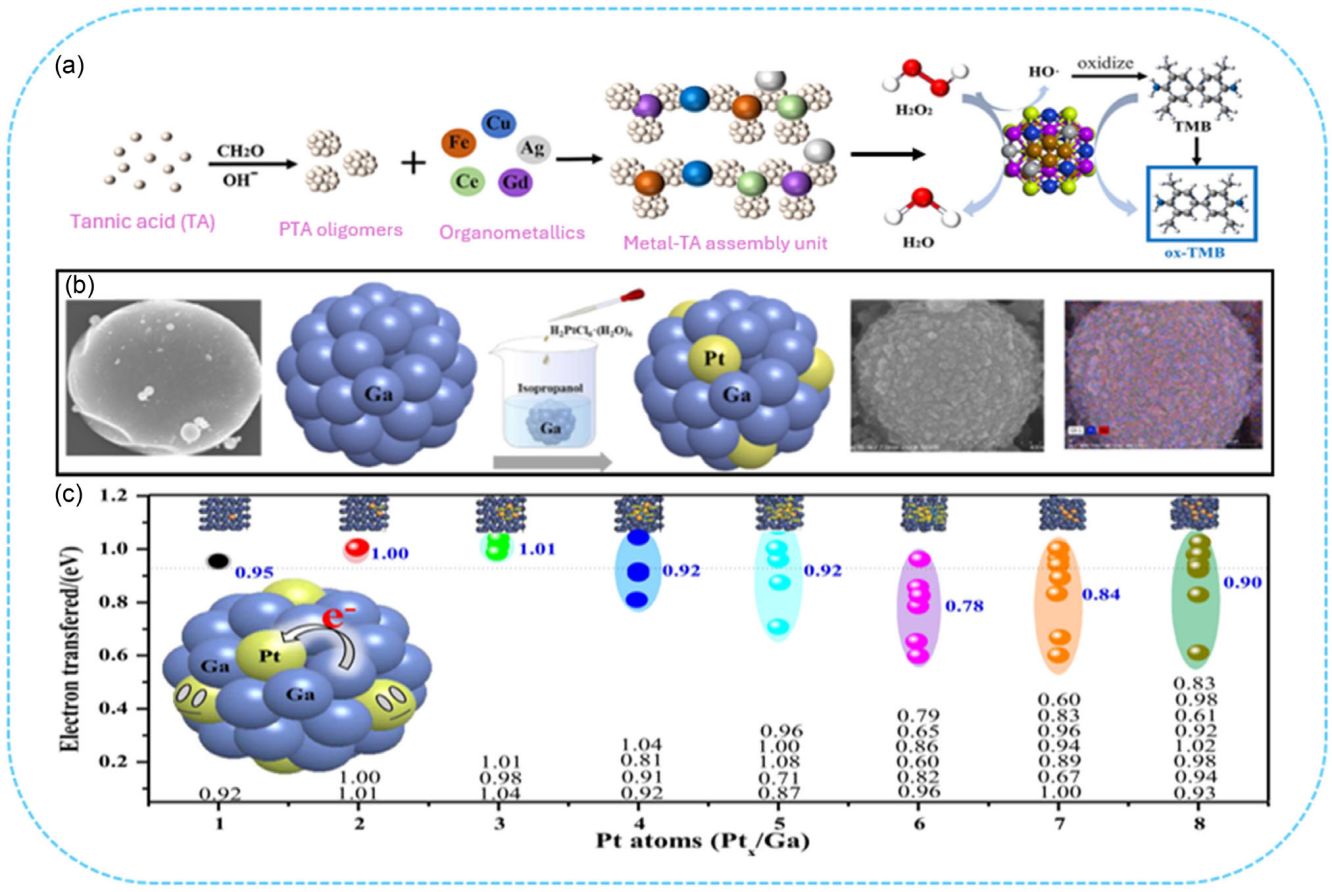
High entropy materials: a) schematic illustration of synthesis of FeCuAgCeGd‐HEAzyme and mimicking the catalytic activity of POD enzyme, enabling the colorimetric detection of DA through H_2_O_2_‐mediated oxidation of TMB. Reproduced with permission from ref. [113] Copyright 2024, Elsevier. Liquid metal catalyst: b) scheme of ultrasonication‐assisted synthesis of Pt SAs in Ga LM for hydrogenation fuel production, with SEM/EDX confirming Pt dispersion on the Ga LM. c) Computational calibration of the electron charge transfer of different Pt_*x*_ SACs in the Pt/Ga catalyst matrix. (b,c) Reproduced with permission from ref. [116] Copyright 2025, Elsevier.

High‐entropy single‐atom catalysts (HESACs) are an emerging class of catalysts that combine multiple metallic elements, typically more than five, in near‐equimolar ratios to create a high‐entropy matrix. This matrix stabilizes isolated metal atoms through strong coordination with surrounding atoms and effectively prevents their migration or aggregation under reaction conditions.^[^ ^109^ ^]^ The high configurational entropy not only enhances structural stability but also introduces a diverse range of electronic environments, enabling tunable adsorption energies and synergistic catalytic effects than the conventional SACs. Based on the choice of support and structural design, HESACs can be broadly categorized into oxide, carbide, nitride, alloy, and 2D based materials.^[^ ^110^, ^111^ ^]^ For instance, Chen et al.^[^ ^112^ ^]^ synthesized FeCoNiRu‐HESACs on nitrogen‐doped graphene through two‐step pyrolysis method. From the theoretical and experimental findings revealed that Fe, Co, Ni, and Ru metal atoms can synergistically modulate the catalytic activity of the Fe site in FeCoNiRu‐HESAC system via nonbonding effect. Peng et al.^[^ ^113^ ^]^ developed a high entropy alloy nanozyme (HEAzyme) based colorimetric sensor by synthesized an ultrafine FeCuAgCeGd HEAzyme using formaldehyde assisted metal‐ligand crosslinking method (**Figure** **13**a). Spherical metal‐ligand frameworks were prepared using tannic acid, formaldehyde, and polyphenol oligomers as cross‐linking agents, into which five metallic elements (Fe, Cu, Ag, Ce, and Gd) were incorporated. After calcination at 900 °C for 3 h, the resulting FeCuAgCeGd‐HEAzyme exhibited peroxidase (POD)‐like catalytic activity, oxidizing colorless 3,3′,5,5′–tetramethylbenzidine (TMB) into blue oxTMB in the presence of H_2_O_2_, enabling colorimetric detection of dopamine (DA). DFT calculation verified the POD like catalytic activity of FeCuAgCeGd HEAzyme in oxidizing colorless TMB to blue oxTMB and facilitate on‐site detection of DA.

HESACs provide a unique multi‐elemental environment that strongly stabilizes isolated atoms, prevents sintering or agglomeration under harsh conditions, and enables tunable adsorption energies and synergistic catalytic effects. These characteristics translate to high catalytic activity and broad applicability across fields such as energy conversion and sensing. Additionally, their versatile design permits integration with various supports, including oxides, carbides, nitrides, alloys, and 2D materials. Despite these advantages, HESACs face significant challenges, including difficulties in characterizing multiple active sites, limited understanding of complex multi‐metal interactions within high‐entropy matrices, and scalability issues that hinder large‐scale practical deployment.

### 4.5 SACs Based on Liquid Metals

Liquid metals (LMs) or their alloys, which remain in a liquid state at or near room temperature due to their inherently low melting points, represent a unique class of materials that combine the deformability of liquids with the conductivity of metals. Typical examples include mercury (Hg), gallium (Ga), cesium (Cs), rubidium (Rb), francium (Fr), and various Ga‐based alloys. These materials naturally exhibit high electrical and thermal conductivity, low viscosity, and high surface tension. Wu et al.^[^ ^114^ ^]^ synthesized Cu‐embedded gallium alloy liquid‐metal catalyst (Cu‐LMC) for methane conversion. In situ XPS and ab initio molecular dynamics simulation revealed Cu active sites enhanced the formation of Cu‐O‐Ga coordination. Thus, preserve the self‐adaptive property of Cu‐LMC and improve the overall methane conversion process. In liquid‐metal single‐atom catalysts (LMSACs), catalytically active metal atoms are dispersed either on the surface or within the liquid metal matrix.^[^ ^115^ ^]^ Unlike conventional solid‐supported SACs, the liquid metal functions as a dynamic and self‐regenerating support that enhances atomic dispersion, improves catalyst stability, and facilitates efficient mass and charge transfer. Moreover, the molten and flexible nature of the LM matrix provides a tunable coordination environment for the single atoms while mitigating high‐temperature sintering or aggregation, thereby overcoming one of the key limitations of traditional SACs. Zhu et al. decorated Ga LM system with Pt SACs via solvent assisted self‐assembly technique for the hydrogenation of stearic acid, achieving efficient fuel production. SEM and EDX mapping images highlight the homogeneous distribution of Pt SAs on the Ga LM (Figure 13b). Computational modeling revealed a synergistic effect of Pt SACs within the Ga matrix wherein modulation of the d‐band electronic structure (Figure 13c) and evidently participate in charge transfer interaction. These electronic tuning significantly enhanced catalytic activity, thereby enables the sustainable conversion of biomass waste into valuable fuel and chemicals.^[^ ^116^ ^]^

Overall, LMSACs include a dynamic and self‐healing matrix that stabilizes isolated metal atoms, suppresses sintering, and provides tunable electronic environments for optimized catalytic performance. Nevertheless, key challenges remain, including the limited availability and high cost of certain liquid metals, difficulties in precisely controlling atomic dispersion and coordination within a fluid matrix, potential toxicity issues (e.g., Hg‐based systems), and the absence of scalable synthesis protocols. Ongoing research is actively addressing these limitations, and LMSACs are expected to play an increasingly important role in advancing next‐generation catalytic technologies across diverse applications.

## 5 SACs Based Materials for Sensor

SACs have emerged as promising hybrid materials for enhanced sensor performance, offering exceptional selectivity and sensitivity. This is primarily attributed to the unique catalytic properties of isolated metal atoms anchored on support matrices, which facilitate efficient and selective detection of target analytes. The advantages of using SACs over conventional nanoparticles lie in the abundance of active sites, which maximizes the exposure of metal atoms. This is further enhanced by the coordination with the support matrix, reducing agglomeration and minimizing susceptibility to ion migration compared to nanoparticles‐based catalysts. In recent decades, SACs have been extensively studied for various sensing applications, targeting a broad range of fields, including environmental monitoring, biosensing, gas sensing, and even biomedical applications.

### 5.1 Environmental Monitoring

**Figure 14:**
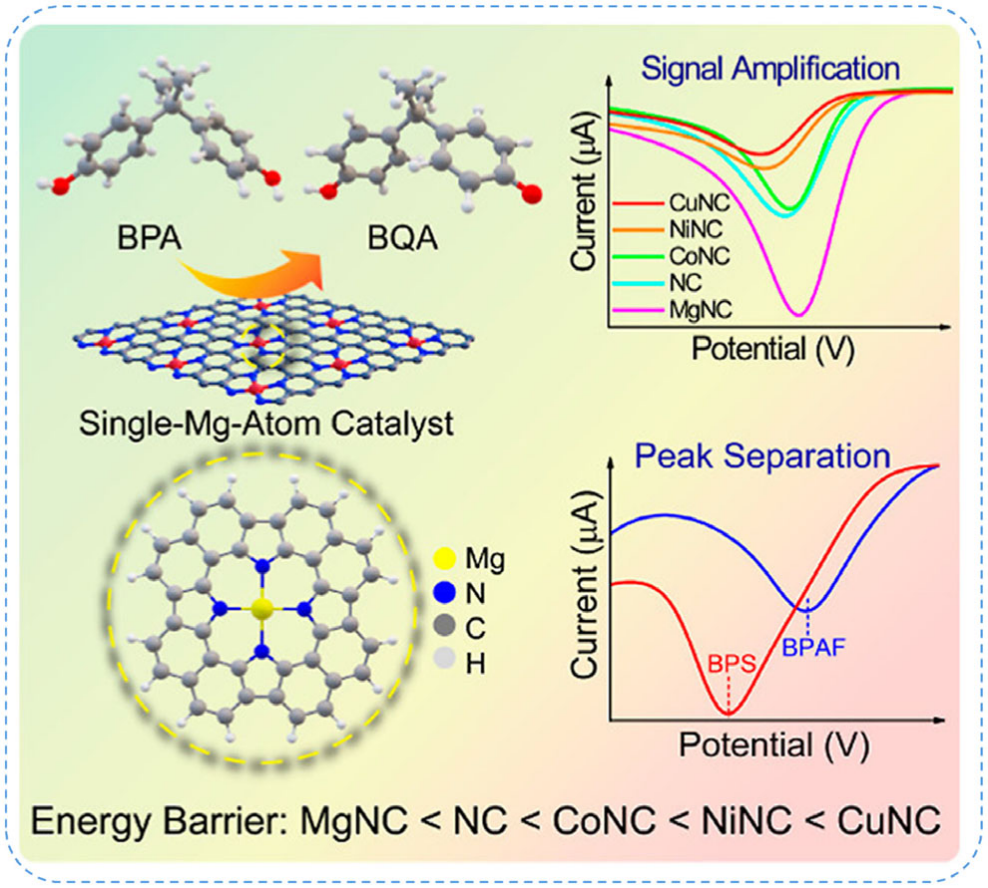
Schematic illustration of single‐Mg‐atom catalyst for sensing applications of BPA in a wide linear range of 0.2 to 5 μM, with a sensitivity of 0.303 μA μM^−1^ and LOD of 0.24 μM. Reproduced with permission from ref. [118] Copyright 2024, American Chemical Society.

Environmental pollutants monitoring through SACs also remains to be in current trend for easy detection of pollutants in water either through advanced oxidation process or other sensing approaches.^[^ ^117^ ^]^ Wu et al. developed Mg‐, Co‐, Ni‐, and Cu‐based SACs supported on NC surfaces for the sensing of bisphenol A (BPA), as shown in **Figure** **14**. HAADF‐STEM imaging confirmed atomically isolated metal centers, while XPS and EXAFS confirmed the formation of distinct M‐N_4_ coordination motifs. Variations in electronic structure tuned by the coordination motifs dictated catalytic activity, with the order of MgNC < NC < CoNC < NiNC < CuNC. Among these, the Mg SACs on NC exhibited the best sensitivity and selectivity for BPA sensing, covering a linear range of 0.2 to 5 μM, with a limit of detection (LOD) of 0.24 μM and a sensitivity of 0.303 μA μM^−1^.^[^ ^118^ ^]^

The bioaccumulation of heavy metals such as arsenic (As), Hg, lead (Pb), and chromium (Cr) from groundwater poses significant health risks even at trace concentrations, underscoring the need for sensitive monitoring. Yao et al. developed a sensor by constructing Fe SACs on NC, functionalized onto a solution‐gated graphene transistor. XANES and EXAFS analyses revealed well‐defined Fe‐N_4_ coordination geometry, which strongly modulated electron density at Fe centers, suppressing aggregation and enhancing electron transfer. And enhanced detection performance for Hg^2+^ ions, offering a linear detection range of 30 nM to 3 μM, a LOD of 1 nM, and a rapid response time of just 2 s.^[^ ^119^ ^]^ Li et al. developed Pt SACs anchored onto MoS_2_ surfaces for the detection of arsenic in its trivalent state (As^3+^), which, along with its other valence states (As^0^ and As^5+^), poses serious health hazards to humans. Aberration‐corrected STEM and XPS spectra confirmed atomically dispersed Pt coordinated via Pt‐S bonds within the MoS_2_ lattice. These Pt‐S motifs provided strong anchoring while facilitating As‐O bond interactions with the analyte, which enhanced charge transfer efficiency. The device achieved a high sensitivity of 3.31 μA ppb^−1^ across a linear detection range of 0.5 to 12 ppb, demonstrating its effectiveness for trace‐level detection of arsenic in aqueous environments.^[^ ^120^ ^]^ Mao et al. developed a colorimetric sensor using Fe SACs supported on NG, for the detection of hexavalent chromium (Cr^6+^) which mimic peroxidase‐like enzymatic catalytic activity. XPS, EXAFS, and DFT simulations identified Fe‐N_4_ active centers as mimics of peroxidase enzymes. These motifs enabled efficient electron transfer and substrate activation. In this system, tetramethylbenzidine (TMB) acts as the chromogenic substrate, while 8‐hydroxyquinoline (8‐HQ) serves as both a chelating and inhibitory agent. The presence of Cr^6+^ facilitates the oxidation of TMB, leading to a measurable decrease in the characteristic blue coloration. The sensor demonstrated a linear detection range from 30 nM to 3 μM, with a LOD of 3 nM and exhibited excellent selectivity against various interfering species.^[^ ^121^ ^]^ **Table** **2** summarizes the performance of SACs‐based sensors in environmental monitoring, highlighting their high sensitivity and detecting specific targets at ultra‐low concentrations, with wide linear detection ranges for real‐world applications. In summary, SAC‐based sensors show great promise for environmental monitoring by enabling highly sensitive and selective detection of pollutants such as BPA and toxic heavy metals (As, Hg, Pb, and Cr) in water. Through tailored coordination motifs and support engineering, these systems achieve ultra‐low detection limits, rapid response times, and strong selectivity, highlighting their potential for real‐world water quality assessment and pollution control.

**Table 2:** Summary of environmental monitoring performance of diverse SACs supported by coordinative materials, focusing on sensitivity and detection limits.

SAC	Supporting material	Analyte	LoD	Linear range	Advantage	Disadvantage	Ref
Mg	NC	Bisphenol A	240 nM	0.2–5 μM	Earth‐abundant, high selectivity due to Mg‐N_4_ motifs	Moderate sensitivity compared to transition‐metal SACs	[118]
Fe	NC	Hg (II)	1 nM	0.03–3 μM	Fe‐N_4_ coordination enhances electron transfer, fast response	Sensitive to pH fluctuations; may suffer from long‐term instability	[119]
Pt	MoS_2_	As (III)	–	0.5–12 ppb	Pt‐S coordination provides ultra‐trace detection and high selectivity	Pt is costly and less earth‐abundant, limiting scalability	[120]
Fe	NG	Cr (VI)	3 nM	0.03–3 μM	Fe‐N_4_ sites nanozyme, excellent selectivity	Performance depends on chelating agents	[121]
Ir	CD	Hg (II)	4.4 nM	0.01–10 μM	High dispersion, stable nanozyme activity	Ir is scarce & expensive; synthesis is complex	[168]

Note: NC – nitrogen doped carbon; NG – nitrogen doped graphene; SA Ir‐CDs – single‐atom iridium‐doped carbon dot nanozymes.

### 5.2 Electrochemiluminescence Sensing

ECL combines electrochemical and optical processes, wherein luminophores generated at the electrode surface undergo reduction upon the application of an external voltage. These reduced species subsequently participate in oxidation reactions, producing excited electronic states that emit light as they return to the ground state, enabling sensitive and selective luminescent detection.^[^ ^122^, ^123^ ^]^ Gu et al. developed Fe SACs supported on graphene nanosheets via a pyrolysis method for the sensitive detection of Trolox, a water‐soluble vitamin E analog and potent antioxidant. HAADF‐STEM and XAFS analyses confirmed atomically dispersed Fe atoms coordinated in a Fe‐N_4_ environment within the graphene lattice. In this study, a classic luminol‐based ECL system was employed, using H_2_O_2_ as a co‐reactant. The presence of H_2_O_2_ facilitated the generation of reactive oxygen species, including superoxide (O_2_^•−^) and hydroxyl (•OH) radicals, which promoted the oxidation of luminol, thereby amplifying the ECL signal. The sensor demonstrated effective detection of Trolox over a broad linear range from 0.8 μM to 1 mM.^[^ ^124^ ^]^ Zhu et al. fabricated crystalline graphitic carbon nitride nanorods integrated with Co SACs as the catalytic sensing material for the detection of miRNA‐222, a biomarker closely associated with diabetes, as well as kidney and pancreatic cancers. XPS and EXAFS studies verified the atomic dispersion of Co with Co‐N coordination motifs. This sensing strategy combined hybridization chain reaction with catalytic hairpin assembly to amplify the detection signal, while interaction with Au electrodes, Au‐S bonds were formed, imparting luminescent properties to the system. In the presence of persulfate (S_2_O_8_^2−^) as a co‐reactant, a redox reaction was triggered, generating highly reactive sulfate radicals (SO_4_^•−^), which significantly enhanced the ECL signal for sensitive miRNA‐222 detection with the linear range of 100 amol L^−1^–1 nmol L^−1^ and LOD of 40 amol L^−1^.^[^ ^125^ ^]^ Bushira et al. developed Fe‐ and Co‐ dual SACs anchored on the N‐doped graphene (NG) with luminol capped Ag nanoparticles for detecting PSA. STEM and XPS characterization confirmed dual Fe‐N and Co‐N coordination environments, which synergistically enhanced active site density and electron transfer. The ECL intensity was intensified 677 times compared to the base catalysts with a linear range of 1 fg mL^−1^−10 μg mL^−1^ and a lower LOD of 0.98 fg mL^−1^.^[^ ^126^ ^]^ **Table** **3** provides an overview of SACs‐based sensors, facilitated by luminophore‐mediated emission, predominantly constructed with carbon‐based supporting materials, emphasizing their ultra‐low detection limits for biomarkers and heavy metal ions detection. Altogether, SAC‐based ECL sensors offer exceptional sensitivity and selectivity by leveraging strong metal‐support interactions and radical‐driven luminol or persulfate systems. Recent advances demonstrate their effectiveness in detecting diverse targets such as antioxidants, cancer‐related miRNAs, and protein biomarkers at ultra‐low concentrations, underscoring their potential for clinical diagnostics and biomedical applications.

**Table 3:** Comparative analysis of SACs‐based ECL with ultra‐low detection limit for biomarkers and heavy metal ions.

SAC	Supporting material	Analyte	LoD	Linear Range	Advantage	Disadvantage	Ref
Fe‐Co	NG	PSA	9.8 × 10^−7^ nM	1 × 10^−6^–10 000 ng mL^−1^	Dual‐metal synergy; high conductivity from NG; ultralow LoD	Complex synthesis; potential long‐term biocompatibility concerns	[126]
Fe‐P	N/C	AA	0.03 nM	0.001–100 μM	Stable Fe‐P coordination; cost‐effective; good catalytic activity	Possible interference from structurally similar molecules	[169]
Fe‐Au	SiO_2_	Hg(II)	0.13 nM	0.01–0.5 nM	Strong affinity of Au for Hg; SiO_2_ enhances dispersion	Narrow linear range; costly Au loading	[23]
Co	cry‐CNN	microRNA‐222	40 amol L^−1^	0.0001–1000 pmol L^−1^	Excellent catalytic activity from Co–N; high specificity with cry‐CNN	cry‐CNN synthesis is less scalable; stability issues in complex matrices	[125]
Fe	N/C	PSA	0.62 pg mL^−1^	0.001–100 ng mL^−1^	Simple synthesis; low cost; wide detection range	Lower sensitivity compared to dual‐atom or HE‐SAC systems	[170]
HE‐SAC	Lum‐AgNPs	miRNA‐21	15.6 aM	100 aM–1 nM	Exceptional sensitivity from multimetal synergy; stable ECL amplification	Complex fabrication; limited reproducibility at large scale	[171]

Note: NG – nitrogen doped graphene; Fe‐N/P‐C – heteroatom iron and phosphorous doped graphitic carbon nitride; cry‐CNN – crystalline graphitic carbon nitride nanorods; NC – nitrogen doped carbon; Lum AgNPs – luminol‐functionalized silver nanoparticles; HESAC – high‐entropy single‐atom catalyst (M = Fe, Co, Cu, Ni, and Mn).

### 5.3 Gas Sensing

**Figure 15:**
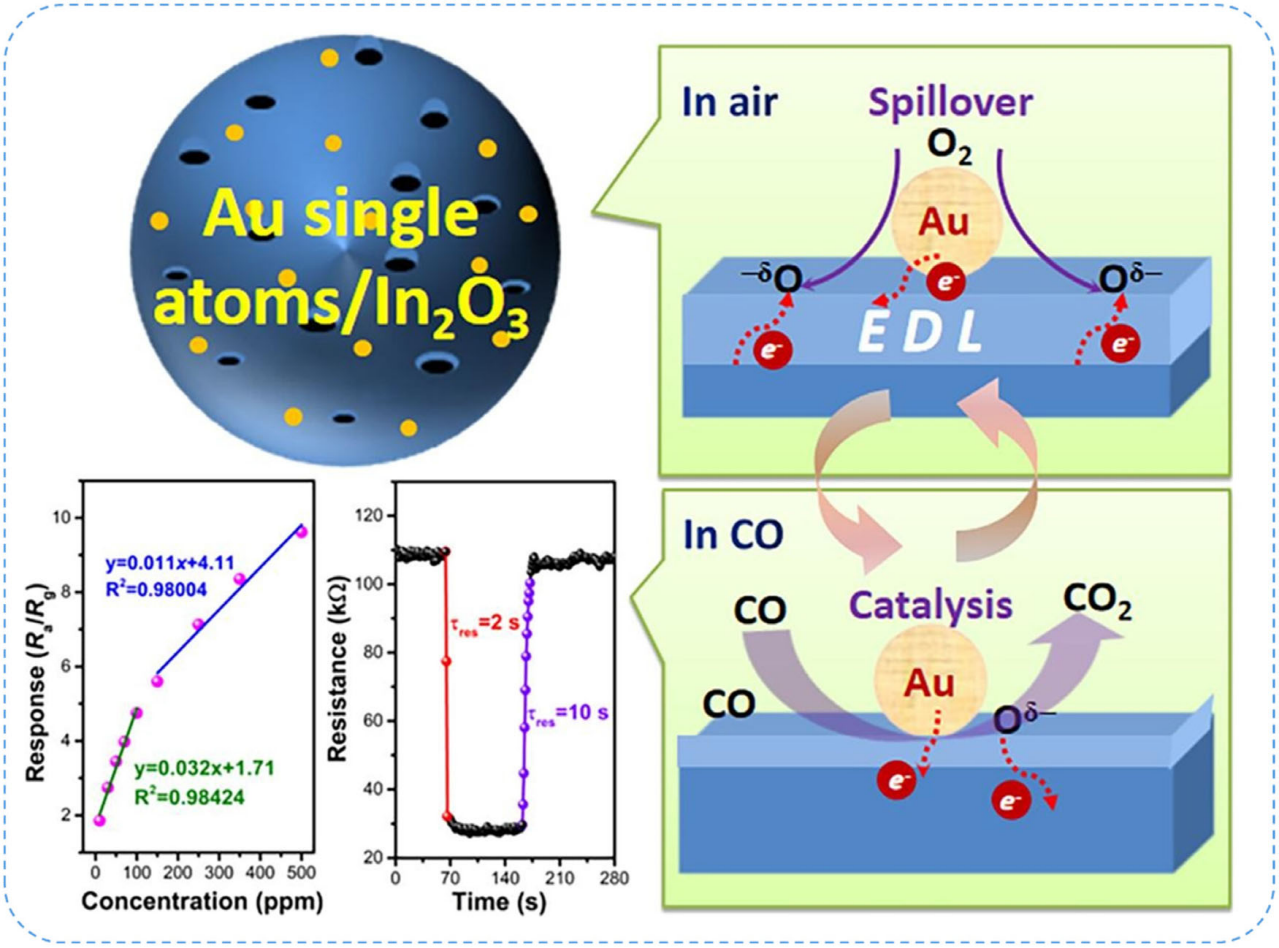
Schematic illustration of In_2_O_3_ porous nanospheres decorated with Au SAs for ultrafast and highly sensitive CO gas detection, achieving a rapid response time of 2 s at 70 ppm, a linear detection range of 10–100 ppm, and an ultra‐low detection limit of 0.032 ppm. Reproduced with permission from ref. [135] Copyright 2023, Elsevier.

Toxic gases released from industrial emissions, transportation, flammable substances, and explosives pose significant and hazardous health risks. In response, the growing integration of Internet of Things (IoT) technologies and human–machine interfaces have spurred intense interest in developing practical gas sensors with low power consumption and enhanced intelligence, often incorporating artificial neural networks. In many gas sensing mechanisms, synergistic interaction between oxygen vacancies and SACs plays a critical role, leading to improved selectivity and significantly reduced response times by facilitating efficient gas adsorption and charge transfer processes.^[^ ^80^, ^81^, ^127^ ^]^ Xue et al. developed Au SACs anchored on a ladder‐like ZnO surface for the effective detection of NO_2_ gas. HAADF‐STEM and XPS analyses confirmed atomically dispersed Au coordinated with oxygen vacancies on ZnO, which enhanced NO_2_ adsorption. Operating at 150 °C, the sensor demonstrated a broad detection range from 12.6 to 300 ppb, with a low LOD of just 1 ppb.^[^ ^128^ ^]^ Rong et al. demonstrated a strategy to sense methanol using Pt SACs supported on porous Ag‐LaFeO_3_@ZnO forming core‐shell structure. XAFS studies revealed Pt‐O coordination within the oxide matrix, which stabilized Pt atoms. On comparing the catalytic activity both with and without Pt SACs the catalytic sensing activity exceeds by 6.68 times compared to base support. The LOD value was found to be 3.27 ppb intimidating the finest design of the catalyst to adsorb methanol gas.^[^ ^129^ ^]^ Sun et al. developed multi‐heterojunctions by sequentially loading SnO_2_ nanorods (SnO_2_ NRs) and Pt SACs on silicon carbide nanosheets (SiC NSs). In this architecture, both SnO_2_ NRs and SiC NSs acted as support matrices for the Pt SACs, creating a heterocatalytic system (Pt SACs@ SnO_2_ NRs@SiC NSs) for the sensitive detection of ethanol. When exposed to ethanol gas at 350 °C, the sensor demonstrated a linear detection range of 10 to 1000 ppm, with impressive response and recovery times of 14 and 20 s, respectively. The system exhibited excellent gas sensing performance with a high sensitivity of 119.75, alongside a shorter response time compared to other sensors, highlighting its superior detection capabilities for ethanol.^[^ ^130^ ^]^ Liu et al., reported on SO_2_ sensing using Ni SACs bonded to SnO_2_ NRs surfaces, where XPS and XAFS confirmed Ni‐O coordination motifs. The response value for SO_2_ sensing is 48 at 20 ppm with a lower LOD of 100 ppb.^[^ ^131^ ^]^ Li et al. decorated Fe_2_O_3_ with Pt SACs forming through the conventional approach of atomic dispersion on the solid catalyst surface. XAFS confirmed Pt‐O coordination, enhancing ethanol sensing was found to be 102.4 exhibiting good selectivity being promoted by the higher valent Pt SACs and better adsorbing capacity of ethanol gas.^[^ ^132^ ^]^ Koga et al. fabricated Co_3_O_4_ nanoparticle film with atomically dispersed Pd SACs for effective sensing of H_2_ gas. In this study, the synthesized Co_3_O_4_ with homogeneous dispersion of Pd atoms forms Pd‐O and Pd‐O‐Co coordination and analyzed by STEM and EXAFS techniques. Upon laser ablation, the Co‐Pd forms an alloy prompting to collect ionosorbed oxygen with the redox conversion of Pd^4+^ to Pd^2+^ aiding to sense H_2_ gas.^[^ ^102^ ^]^ Zhang et al. developed Pt SACs on Fe_2_O_3_ NSs using atomic layer deposition strategy for H_2_ sensing and XAS confirmed Pt‐O‐Fe coordination. The Pt‐Fe_2_O_3_ oxygen rich material aids to sense H_2_ with a lower response time of 2 s in the linear range of 26.5–50 ppm which is 17 times higher than that of the base catalyst and holds lower LOD of 86 ppb.^[^ ^133^ ^]^ Ye et al. proposed a conceptual proof for sensing CO using Pd SACs on the TiO_2_ coordinated surface. The CO sensing ranges from 0.1 to 1000 ppm with a response time of 28 s and a better selectivity compared to 12 interferent gas molecules.^[^ ^134^ ^]^ Li et al. have developed porous In_2_O_3_ nanospheres with Au SACs for CO sensing (**Figure** **15**). STEM and XPS confirmed Au‐O coordination at vacancy sites. In this approach, the sensing performance was assessed in 360 °C and the response time was 2 s for target at 70 ppm of CO, while the sensor linear range was from 10 to 100 ppm with a lower sensitivity of 0.032 ppm.^[^ ^135^ ^]^ Shin et al. have developed a heterojunction catalyst of exfoliated carbon nitride with SnO_2_ being stabilized with Pt SACs. EXAFS analyses revealed Pt‐N/O coordination motifs, which promoted formaldehyde (HCHO) detection. The HCHO gas testing was performed in the range of 5 ppm–50 ppb at 275 °C exhibits a lower response time of 33.9 and a catalytic stability was found until 170 h of continuous sensing.^[^ ^136^ ^]^ **Table** **4** summarizes the role of atomically dispersed metal active sites within metal‐oxide support materials, highlighting their effectiveness in real‐time monitoring and detection of toxic gases. In remarks, SAC‐based gas sensors achieve high sensitivity, selectivity, and fast response times by exploiting strong metal‐support interactions and oxygen vacancy engineering. Systems incorporating Au, Pt, Pd, Ni, and other SACs effectively detect toxic and flammable gases at ppb levels, including NO_2_, methanol, ethanol, SO_2_, H_2_, CO, and formaldehyde. These advances position SACs as promising candidates for next‐generation, low‐power, and durable gas sensing devices in environmental and industrial applications.

**Table 4:** Summary of SACs‐based gas sensor performance with detection sensitivity and response characteristics.

SAC	Supporting material	Analyte	LoD	Linear range	Advantage	Disadvantage	Ref.
Au	ZnO	NO_2_	1 nM	10–300 ppb	Low detection limit; strong Au–O coordination with oxygen vacancies	Operates at elevated temperature; costly noble	[128]
Ni	SnO_2_	SO_2_	100 ppb	–	Earth‐abundant, low‐cost metal; Ni–O bonding enhances SO_2_ adsorption	Moderate sensitivity and selectivity	[131]
Pt	Fe_2_O_3_	H_2_	86 ppb	26.5–50 ppm	Pt‐O‐Fe coordination ensures fast electron transfer; high sensitivity	Noble‐metal cost; narrow linear range	[133]
Au	In_2_O_3_	CO	–	10–500 ppm	Good selectivity via Au‐O vacancy sites; stable dispersion	Higher detection limit; stability issues	[135]
Sn	Fe_2_O_3_	NO_2_	10 ppb	–	Low‐cost Sn; defect‐rich Fe_2_O_3_ stabilizes active sites	Limited linear range; long‐term stability not well‐established	[172]
Pt	In_2_O_3_(NG)	HCHO	8.4 ppb	0.1–100 ppm	Very low LOD; Pt‐N/O coordination improves sensitivity and durability	High synthesis complexity; expensive Pt	[173]

Note: Sn‐Fe_2_O_3_ – tin incorporated porous iron oxide; HCHO – formaldehyde; Pt‐In_2_O_3_(NG) – platinum on MOF‐derived In_2_O_3_ via nitrogen doped graphene.

### 5.4 Biosensing

Biosensing possesses a higher demand for catalytic characterization specifically for in vivo or in vitro biomolecules and biomarker detection. In the current decade, SACs are trendily focused on biosensing via modulating electron transfer kinetics and ion transport behavior. Zeng et al. synthesized Pt SACs supported on the NiCo layered double hydroxide (LDH)/NG to sense glucose non‐enzymatically. The loading concentration of Pt SACs depends on the concentration of Co atoms on the LDHs since they generate anchoring support for the SACs on the LDHs. Advanced characterization by HAADF‐STEM and XAFS confirmed the uniform dispersion of Pt atoms and the presence of Pt‐O/Co coordination motifs, which ensured stable atomic binding. Upon applying for glucose sensing, the catalyst was prone to exhibit lower oxidation potential of 0.440 V with better sensitivity of 273.78 μA mM^−1^·cm^−2^. The developed Pt/NiCo LDH/NG possesses stronger binding affinity for glucose with a synergism of Pt SACs, Co doping, and NG.^[^ ^137^ ^]^ Song et al. synthesized N‐doped carbon aerogels (NCA) embedded with Co SACs through pyrolytic conversion of carbon‐rich biomass hydrogels. The resulting 3D NCA framework not only promoted efficient mass transport but also stabilized atomically dispersed Co atoms through well‐defined Co‐N_4_ coordination motifs, as confirmed by EXAFS and XPS analyses. These coordinatively unsaturated Co sites acted as the primary active centers, enabling rapid electron transfer and efficient catalytic oxidation of glucose. In sensor measurements, NCA‐Co SACs detect glucose over a linear range of 0.5 μM–6 mM with a LOD of 0.1 μM, along with real‐time assessment of glucose from artificial saliva and human serum samples.^[^ ^138^ ^]^ Wu et al. fabricated Fe SACs impregnated with similar NCA, thereby demonstrating its applicability with both colorimetric and electrochemical sensing of glucose. For electrochemical sensing, Fe–N coordination motifs facilitated the apparent electrocatalytic oxidation of glucose was assessed at 0.35 V over a linear range of 2–2000 μM with an LOD of 0.5 μM in alkaline environment. For colorimetric sensing approach, O‐phenylenediamine was oxidized in H_2_O_2_ environment mimicking to enzymatic approach of glucose detection with a linear range of 0.1–4 mM having an LOD of 3.1 μM.^[^ ^139^ ^]^

**Figure 16:**
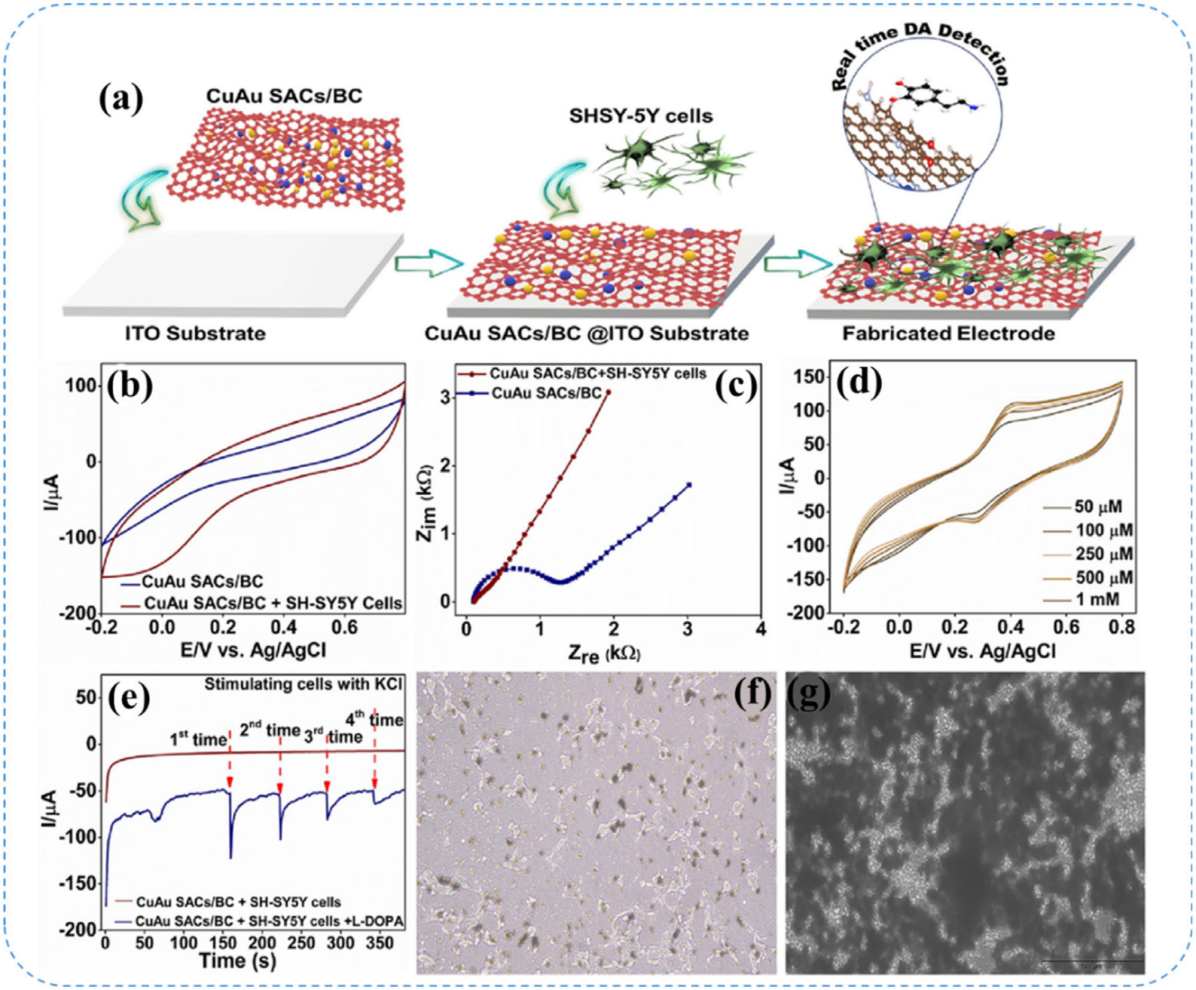
Real‐time detection of dopamine (DA) released from neuronal cells. a) Schematic illustration of the fabrication of CuAu SACs/BC‐based real time sensing of dopamine in live cells. b,c) CV and EIS signals, respectively, of CuAu SACs/BC and CuAu SACs/BC with SH‐SY5Y cells grown electrodes. d) CV results of various concentration of DA from 50 μM to 1 mM using CuAu SACs/BC + SH‐SY5Y modified electrode. e) Amperometric detection of DA released from SH‐SY5Y when stimulated with 120 mM KCl at different time intervals. f) Microscopic image of SH‐SY5Y cells cultured on the surface of the CuAu SACs/BC fabricated electrode for 24 h. g) Microscopic image of SH‐SY5Y cells after real time sensing of DA. Reproduced with permission from ref. [142] Copyright 2023, Elsevier.

Focusing on neurotransmitter sensors, particularly, dopamine (DA), a key biomarker for such as Parkinson's disease diagnosis, Sun et al. developed a highly sensitive sensor based on Ni SACs anchored onto MoS_2_ nanosheets. Structural studies confirmed that Ni atoms were axially coordinated to S sites in MoS_2_, enhancing the exposure of active Ni centers and optimizing electron density for efficient DA oxidation. This sensor demonstrated ultra‐sensitive DA sensing over 1 pM in neutral environment, 1 pM in bovine serum samples and 100 pM in artificial urine.^[^ ^140^ ^]^ Liu et al. developed porphyrin zirconium‐based MOF (HPCN‐222) as supports for Pt SACs, simultaneously forming a hollow 3D architecture that effectively anchors Pt atoms. HAADF‐STEM, XAFS studies confirmed the formation of Pt‐N_4_ coordination environments. This design enhances the catalytic activity and electrochemical stability of Pt SACs, enabling highly sensitive detection of levodopa (L‐DOPA). The linear range for L‐DOPA sensing was assessed from 0.1 to 130 μM holds a sensitivity of 0.1066 μA μM^−1^. To extend the study toward real‐time applications, human serum samples were analyzed, demonstrating consistent electrochemical stability over 150 days when stored at 4 °C, along with a satisfactory recovery rate.^[^ ^141^ ^]^ Chellasamy et al. designed and fabricated dual SACs based on Cu and Au supported on bioinspired chitosan‐derived carbon (BC) extracted from barnacles, as illustrated in **Figure** **16**a. The pyrolytic incorporation of Cu and Au SAs into the carbon matrix formed stable Cu‐N and Au‐C coordination motifs. The CV and EIS analyses confirmed the successful culture of SH‐SY5Y cells on the CuAu SACs/BC surface, indicated by an enhanced current response compared to the bare catalyst (Figure 16b,c). Real‐time noninvasive DA sensing was performed in geriatric plasma samples and neuronal cells of SH‐SY5Y with a lower LOD of 50 nM (Figure 16d,e) and 13.5 nM for geriatric plasma samples with 1.6% RSD recovery rate.^[^ ^142^ ^]^ Microscopy and cellular analysis revealed strong cell adhesion on the CuAu SACs/BC electrode and morphological changes after sensing, indicating effective DA release. These findings highlight the catalyst's high catalytic activity, improved conductivity, and excellent biocompatibility for electrochemical DA detection in both biofluids and cellular environments (Figure 16f,g).

Hu et al. developed an electrochemical sensor for uric acid (UA) detection based on Co SACs coordinated with NG matrices, mimicking enzymatic activity. Advanced characterization by HAADF‐STEM and XAFS confirmed atomically dispersed Co sites coordinated in a Co‐N_4_ environment. These isolated Co centers acted as the primary active sites for UA oxidation, enabling a low LOD of 33.3 nM across a wide linear range of 0.4 to 41.950 μM. Real‐time analysis using human serum samples demonstrated a high recovery rate of 98.5%, while long‐term stability test demonstrated retention of 90.5% of its initial electrocatalytic activity after 180 days of storage, indicating excellent stability and reliability of the catalyst.^[^ ^143^ ^]^ Wu et al. prepared Ru SACs supported on carbon nanocages derived from ZIF‐8. Structural characterization through EXAFS and XPS revealed atomically dispersed Ru centers stabilized by Ru‐N coordination motifs within the carbon framework. These Ru‐N sites provided superior catalytic activity toward UA oxidation, with a wide linear detection range of 0.05–4000 μM and lower LOD of 10 nM. When applied to serum sample analysis, the sensor retained 89.5% of its original activity after 20 days of storage with a recovery rate of 96%, indicating good stability and reliability.^[^ ^144^ ^]^ **Table** **5** provides an overview of biomolecule and biomarker detection using atomically dispersed metal active sites within a tailored coordinative environment of carbon materials, highlighting that heteroatom‐based SACs offer enhanced sensitivity compared to homoatom SACs, attributable to the synergistic electronic modulation and optimized binding affinity introduced by heteroatom coordination. Overall, SAC‐based biosensors offer exceptional sensitivity, selectivity, and stability for biomolecule detection, driven by coordination motifs such as M‐N, M‐S, and M‐O that stabilize active sites and enhance electron transfer. Advanced characterization confirms atomic dispersion, while biocompatible supports enable reliable real‐sample applications, underscoring their strong potential in clinical diagnostics.

**Table 5:** Comparative analysis of SACs based biosensor with ultra‐low concentration and detection limit.

SAC	Supporting material	Analyte	LoD	Linear range	Advantage	Disadvantage	Ref.
Co	NCA	Glucose	100 nM	0.5–6000 μM	High sensitivity, wide linear range, good conductivity from NCA support	Less selectivity; moderate stability under long‐term use	[138]
Fe	NCA	Glucose	3100 nM	2–2000 μM	Dual‐mode sensing (electrochemical and colorimetric), simple fabrication	Required alkaline medium for best performance	[139]
Pt	HPCN‐222	L‐DOPA	3 nM	0.1–130 μM	Uitra‐low LOD, stable Pt‐N_4_ coordination, durable in real serum samples	Costly noble metal	[141]
Cu‐Au	BC	DA	200 pM	0.001–100 μM	Dual SAC synergy enhances electron transfer; excellent biocompatibility	Complex synthesis; possible leaching of dual metals in long‐term operation	[142]
Fe‐Mn	NC_etch_	HER2	7.5 pg mL^−1^	0.01–2.0 ng mL^−1^	Bimetal synergy improves selectivity	requires controlled etching & stability issues	[174]

Note: NCA – nitrogen doped carbon aerogel; HPCN‐222 – porphyrin zirconium‐based MOFs; BC – bioinspired chitosan‐extracted carbon; FeMn‐NC_etch_/SAC – iron–manganese ion N‐doped carbon single‐atom catalyst; HER2 – human epidermal growth factor receptor 2.

### 5.5 Photoelectrochemical Sensing

**Figure 17:**
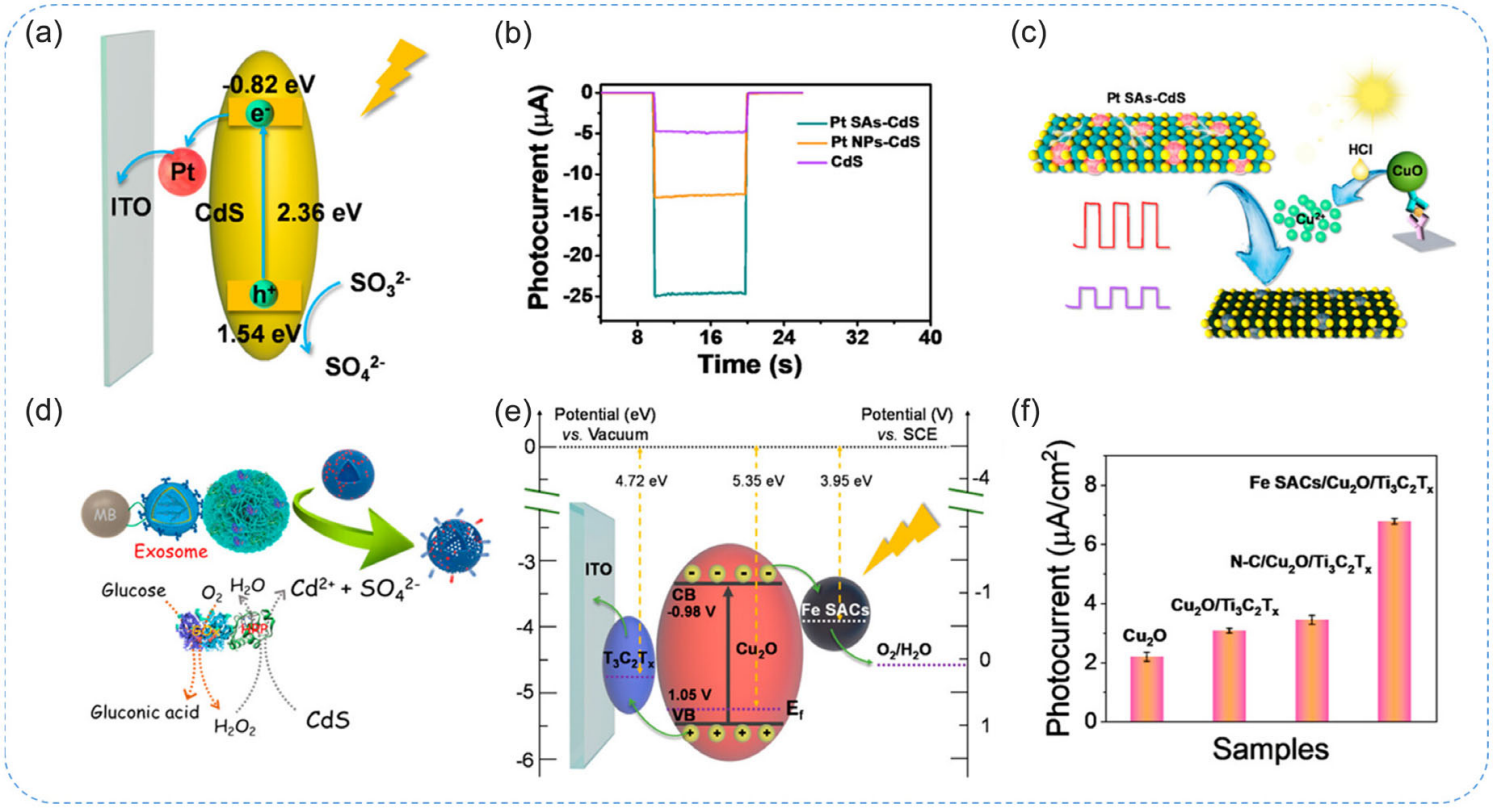
a) Schematic energy band structure of CdS with incorporated Pt SACs, illustrating the modulation of band alignment and enhanced charge carrier dynamics. b) Photocurrent responses of CdS, Pt NPs‐CdS, and Pt SAs‐CdS, exhibit the enhancement in photoelectrochemical performance upon Pt incorporation. c) Schematic illustration of Pt SACs anchored on CdS, illustrating the design strategy for enhanced photoelectrochemical detection of PSA. (a–c) Reproduced with permission from ref. [146] Copyright 2021, American Chemical Society. d) Scheme of the PEC biosensing platform based on enzyme‐modified DNA flowers and Pt SACs–CdS nanostructures for exosome detection. Reproduced with permission from ref. [147] Copyright 2020, Elsevier. e) Schematic energy band structure illustrating the interfacial ORR mechanism on Fe SACs supported by MXenes/CuO_2_ for paraoxon detection. f) Photocurrent responses of CuO_2_, MXenes/CuO_2_, N–C/MXenes/CuO_2_, and Fe SACs/MXenes/CuO_2_ materials shown as a bar graph. (e–f) Reproduced and adapted with permission from ref. [148] Copyright 2022, American Chemical Society.

In the PEC sensing process, a photoactive material is employed to absorb light and generate electron–hole pairs. These charge carriers undergo separation and participate in interfacial redox reactions, which are critical for amplifying the photocurrent signal and enhancing the sensor's sensitivity.^[^ ^145^ ^]^ Qin et al. synthesized heterogeneous Pt SACs on the surface of CdS incorporating CuO nanoparticles as the photocatalytic component for prostate‐specific antigen (PSA) detection. CuO acted as photon generators, while Pt SACs served as catalytic centers coordinated with surrounding Cd‐S ligands, ensuring strong Pt‐S coordination. Characterization by HAADF‐STEM and XAS confirmed atomically dispersed Pt sites, while electrochemical studies verified enhanced charge separation and photocurrent generation. The sensor exhibited a linear detection range of 5 pg mL^−1^–10 ng mL^−1^ with a LOD of 0.92 pg mL^−1^ (**Figure** **17**a–c), and demonstrated real‐time applicability in serum samples, showing a relative standard deviation of 2.5%.^[^ ^146^ ^]^ Zeng et al. developed Pt SACs anchored on hollow CdS nanospheres using a reliable SiO_2_ template‐assisted strategy for exosome model analyte sensing, which could potentially serve as a sensitive biomarker detection method for early‐stage cancer cells. Coordination analysis indicated Pt‐S interactions between Pt atoms and CdS surfaces, which enhanced electron transfer efficiency. Advanced TEM and XPS confirmed the uniform dispersion of Pt atoms, while ECL and PEC characterization demonstrated excellent photoresponse. In this study, horseradish peroxidase (HRP) and glucose oxidase (GO_*x*_) enzymes were employed to encapsulate DNA nanoflowers (Figure 17d), which act as biorecognition elements while simultaneously activating the enzymatic bio etching of exosomes.^[^ ^147^ ^]^ Qin et al. fabricated Fe SACs on the surface of MXene sheets integrated with Cu_2_O for the detection of paraoxon, an organophosphorus pesticide (Figure 17e). To mimic enzymatic activity, the system utilizes HRP, which depresses the photocurrent for enhanced sensing. Additionally, acetylcholine esterase is employed to facilitate a proton‐generating bioreaction for the detection of paraoxon (Figure 17f). XAS confirmed the Fe‐N/O coordination motifs, and electrochemical analyses revealed efficient charge transfer dynamics. The sensor exhibited a linear detection range from 0.5 to 600 ng mL^−1^ with a high reliability of 98.6%.^[^ ^148^ ^]^ Li et al. developed Pt SACs anchored on zinc‐cadmium sulfide (ZCS, Zn_0.5_Cd_0.5_S) nanocrystals, where Pt SAs formed Pt‐S coordination bonds with the sulfide matrix. XANES and EXAFS verified the atomic dispersion of Pt, while photoluminescence and photocurrent studies confirmed efficient electron‐hole separation. This system was designed for the sensitive detection of PSA with a linear range of 0.001–10 ng mL^−1^ and a LOD of 0.22 pg mL^−1^.^[^ ^149^ ^]^ **Table** **6** presents the performance of light‐driven electrochemical reactions based on SAC‐modified materials for detecting diverse analytes, highlighting their potential to meet future practical sensing demands. Notably, SAC‐based PEC sensors stabilized by motifs such as Pt‐S and Fe‐N/O show excellent charge separation and photocurrent amplification, enabling ultrahigh sensitivity and reliability for biomedical and environmental detection.

**Table 6:** Summary of SACs based PEC performance for detecting diverse target analytes.

SAC	Supporting material	Analyte	LoD	Linear range	Advantage	Disadvantage	Ref
Pt	HCdS	Exosome	157 μL^−1^	500–50 000 μL^−1^	High stable Pt sites, strong light absorption, reliable sensitivity	Complex synthesis	[147]
Fe	Cu_2_O‐Ti_3_C_2_T_*x*_	Organo‐phosphorus pesticide	0.08 nM	0.5–600 ng mL^−1^	Excellent selectivity, defect‐rich support enhances binding	Moderate complexity in synthesis, limited large‐scale validation	[148]
Pt	Zn_0.5_Cd_0.5_S	PSA	0.00022 nM	1–10 000 pg mL^−1^	Extremely low LoD, strong synergistic effect	Use of Cd (toxic element), stability concerns under prolonged use	[149]
Co‐Zn	N–C	CAP	0.074 pg mL^−1^	0.0001–100 ng mL^−1^	Earth‐abundant metals, cost‐effective, low LoD	Structural instability, moderate reproducibility	[175]
Fe	Au@p‐COFs	AβO	7.4 fM	10 fM–200 nM	Ultra‐low LoD, excellent selectivity, multifunctional COF support	Complex fabrication, high material cost	[176]

Note: HCdS – hollow CdS nanospheres; CAP – chloramphenicol; Co/Zn–N–C – bimetallic (cobalt and zinc) nitrogen doped carbon; AβO – amyloid‐β oligomer; Au@p‐COFs – gold nanoparticles doped porphyrin‐based covalent organic frameworks (COFs).

### 5.6 AI in Sensors

The integration of machine learning (ML) and AI has recently emerged as a transformative strategy for advancing SACs in sensing applications. The rational design of SACs remains inherently challenging, because their preparation involves complicating steps, including material synthesis and optimization of performance metrics. DFT calculations have been employed for the theoretical prediction of structure‐activity relationships. However, such simulations typically demand substantial computational resources, with high power and time consumption.^[^ ^150^, ^151^ ^]^ To address this limitations, AI particularly ML and neural networks (NNs) have emerged as a powerful methodology for accelerating the discovery and design of SACs. By analyzing large datasets from experimental studies and theoretical calculations, AI can extract key descriptors and establish predictive models to forecast catalytic performance under diverse conditions.^[^ ^152^, ^153^ ^]^ ML algorithms can rapidly identify optimal coordination motifs (e.g., M‐N_4_, M‐S_4_) and catalyst combinations within hours, drastically reducing both time and resource consumption compared to conventional methods.^[^ ^154^, ^155^ ^]^ Moreover, AI can uncover intrinsic relationships between structure and function, enabling theoretical support for rational optimization, and even predict catalyst behavior under unexplored reaction conditions, offering a data‐driven pathway toward the intelligent design of high‐performance SAC‐based sensors.^[^ ^156^, ^157^, ^158^ ^]^

In parallel, AI‐integrated biosensing platforms have been increasingly developed and applied in diverse fields such as food quality and safety, environmental monitoring and clinical applications, owing to their high sensitivity, and advanced data‐processing capabilities. For example, Wang et al. developed a colorimetric sensor based on CuZn‐N bimetallic SAs for the effective detection of bio‐thiols, achieving a detection limit of 1.17 nM. The system was associated with the “YOLO v5 model” algorithm and implemented as a smartphone application (ThiolSense), which employs image segmentation and feature extraction of RGB channels combined with principal component analysis and hierarchical clustering analysis (HCA) to significantly enhance detection accuracy in serum samples.^[^ ^159^ ^]^ In another work, Wei et al.^[^ ^160^ ^]^ prepared different metal doped carbon dots (Fe‐CDs, Mn‐CDs, Cu‐CDs, and Cr‐CDs) integrated with ML algorithms, including HCA, linear discriminant analysis (LDA), artificial neural networks (ANN), K‐nearest neighbors (KNN), decision trees (DT), and support vector machines (SVM), for the selective detection of eight different bio‐thiols, achieving detection limit as low as 40 nM. A notable innovation of the work was algorithm‐fusion strategy, in which an LDA‐SVM cascade model synergistically improving recognition accuracy from 80 to 100%, thereby substantially enhancing detection reliability and paving the way for multiplex sensor platforms. The works clearly demonstrate how coupling SAC‐based nanozymes with advanced algorithms can overcome the challenges of sensitivity, selectivity, and multiplex detection in complex biological environments. Collectively, the convergence of SACs with AI‐driven design principles and AI‐integrated biosensing systems makes a paradigm shift in the field of chemical sensing. These approaches not only accelerate catalyst discovery and rational optimization but also significantly improve sensing accuracy, reliability and throughput. Together, they provide a clear path for moving beyond prototypes toward practical technologies and real‐world applications.

## 6 Summary, Challenges, and Perspectives

In this review, we comprehensively examined the synthesis, characterization, and application of SACs, with a particular focus on their emerging roles in sensing technologies. We first delineated top‐down and bottom‐up synthetic strategies, emphasizing scalable methodologies such as pyrolysis, wet‐chemistry, and templating techniques that facilitate the formation of well‐defined coordination environments while maintaining atomic dispersion. Advanced characterization tools, including XAS, electron microscopy, and DFT, were then discussed for their critical role in elucidating the structure‐activity relationships of SACs. Furthermore, we explored the influence of various support materials such as MOFs, carbon‐based matrices, and MO_*x*_ on the stabilization of SAs and modulation of their catalytic properties. Finally, recent progress in SAC‐enabled sensing platforms was highlighted, encompassing biosensing, environmental monitoring, gas sensing, electrochemiluminescence, and photoelectrochemical detection. In these applications, SACs demonstrate remarkable sensitivity, selectivity, and ultralow detection limits. However, despite these advancements, several critical challenges continue to hinder their practical deployment in real‐world applications. These limitations span from synthesis and stability to device integration and standardization, as outlined below: 1) The synthesis of SACs is challenged by the difficulty of stabilizing isolated atoms under harsh conditions. To prevent aggregation, metal loading is kept below 1 wt%, which limits the density of active sites. High surface energy leads to migration and nanoparticle formation, while precursor impurities further hinder atom isolation and uniformity. 2) Sensing mechanisms in SAC‐based systems are often governed by subtle processes such as charge transfer or weak adsorption events, which are difficult to detect and interpret. Moreover, both the metal centers and the support contribute to the overall activity, complicating the identification of the true active sites. 3) Under electrochemical operating conditions, carbon and oxide supports are susceptible to degradation, such as electrochemical oxidation or dissolution, especially at elevated potentials. These processes can cause the detachment, migration, or aggregation of isolated SAs. Although increasing the graphitization degree of carbon supports can improve their structural and electrochemical stability, it concurrently reduces the number of defect sites and heteroatom dopants that are essential for anchoring SAs, ultimately compromising catalytic performance. 4) The reliance on noble metals like Pt, Pd, or Au limits commercial scalability due to cost and scarcity. Moreover, achieving consistent atomic dispersion at industrial scales remains a major obstacle to widespread adoption. 5) SACs in electrochemical applications often show limited real‐time performance due to inefficient mass transport caused by restricted pore structures and dense catalyst packing, which hinder the rapid diffusion of reactants and products to and from active sites. Achieving optimal performance requires the design of hierarchically porous supports, where micropores provide a high density of active sites, and mesopores/macropores facilitate rapid diffusion of reactants and products. However, precisely engineering such multiscale porosity remains technically challenging but is critical for maximizing catalytic efficiency. 6) Stability under prolonged electrochemical bias or continuous light irradiation remains a critical challenge, as SACs can experience restructuring, aggregation, or support degradation, resulting in performance decline under practical conditions. 7) Integration of SACs into micro and nanoscale devices, including sensors and energy conversion systems, remains underexplored. The challenges lie in ensuring strong interfacial bonding, compatibility with device architectures, and retention of atomic dispersion during operation. 8) The lack of standardized testing protocols across the field hampers reliable comparison of catalytic activity, stability, and selectivity. Establishing universal guidelines and descriptor‐based evaluation frameworks is crucial for scaling SACs from laboratory research toward industrial practice.

SACs offer immense promise in sensing technologies due to their unparalleled atom utilization and well‐defined active sites. Yet, realizing their full potential demands overcoming persistent challenges in synthesis, stability, selectivity, mechanistic understanding, and scalability. Addressing these hurdles requires a multidisciplinary approach that combines advanced materials science, engineering, theoretical modeling, and sustainable manufacturing practices. Future strategies must focus on innovative synthesis methods such as defect engineering, self‐limiting growth, in situ trapping, and rapid annealing to ensure the stable anchorage of isolated atoms while preventing aggregation. Enhancing active site density will rely on eliminating trace contaminants, reinforcing metal‐ligand coordination, and maintaining anchoring sites through inert atmospheres during thermal treatments. To further enhance catalytic performance, microenvironment engineering using heteroatom doping and rational precursor design will be critical in modulating the electronic structure of active sites. In parallel, the development of synergetic catalytic systems such as dual‐atom catalysts and hybrid structures with nanocrystals will enable new reaction pathways and overcome conventional scaling limitations. Ensuring long‐term stability demands the design of robust metal‐support interactions and graphitized support with controlled defect densities, alongside a deeper understanding of degradation mechanisms under operational conditions. Moreover, tailored porosity through self‐assembly techniques using MOFs, COFs, or templated materials will further enhance mass transport and active site accessibility.

Future progress in the integration of AI‐driven platforms with advanced in‐situ and operando characterization methods combined with computational tools such as DFT and ML will be indispensable for real‐time monitoring and rational design, enabling predictive modeling of coordination environments, stability under operating conditions, and high‐throughput screening of optimal active sites. Finally, adopting green synthesis protocols and scalable production methods such as aqueous‐phase synthesis, bio‐templating, and continuous flow systems will be essential for transitioning SACs from the laboratory to commercial applications, ensuring both environmental sustainability and industrial viability.

## Conflict of Interest

The authors declare no conflict of interest.
